# A revision of *Evaniscus * (Hymenoptera, Evaniidae) using ontology-based semantic phenotype annotation

**DOI:** 10.3897/zookeys.223.3572

**Published:** 2012-09-25

**Authors:** Patricia L. Mullins, Ricardo Kawada, James P. Balhoff, Andrew R. Deans

**Affiliations:** 1 Department of Entomology, North Carolina State University, Campus Box 7613, 2301 Gardner Hall, Raleigh, NC 27695-7613 USA; 2 Museu de Zoologia da Universidade de São Paulo. Av. Nazaré, 481, Ipiranga, CEP 04263-000. São Paulo-SP, Brazil; 3 National Evolutionary Synthesis Center, Durham, North Carolina, USA; 4 Department of Biology, University of North Carolina at Chapel Hill, Chapel Hill, North Carolina, USA; 5 Department of Entomology, Pennsylvania State University, 501 ASI Building, University Park, PA 16802 USA

**Keywords:** Anatomy, objectification of morphological descriptions, data accessibility, phenotype, phylogeny, morphology, semantic species description, biodiversity informatics, New World, OWL

## Abstract

The Neotropical evaniid genus *Evaniscus * Szépligeti currently includes six species. Two new species are described, *Evaniscus lansdownei * Mullins, **sp. n.** from Colombia and Brazil and *Evaniscus rafaeli * Kawada, **sp. n.** from Brazil. *Evaniscus sulcigenis * Roman, **syn. n.**, is synonymized under *Evaniscus rufithorax * Enderlein. An identification key to species of *Evaniscus * is provided. Thirty-five parsimony informative morphological characters are analyzed for six ingroup and four outgroup taxa. A topology resulting in a monophyletic *Evaniscus * is presented with *Evaniscus tibialis * and *Evaniscus rafaeli * as sister to the remaining *Evaniscus * species. The Hymenoptera  Anatomy Ontology and other relevant biomedical ontologies are employed to create semantic phenotype statements in Entity-Quality (EQ) format for species descriptions. This approach is an early effort to formalize species descriptions and to make descriptive data available to other domains.

## Introduction 

Deans et al. (2012) recently opined that phenotype data collected by taxonomists, i.e., the natural language character statements found in diagnoses and descriptions, could, if presented in a broadly accessible, searchable manner, be used to address big questions in biology. Other components of the taxonomic process – names, specimens, DNA sequences, images, etc. – are already digitized and therefore contribute to discoveries in other contexts (Patterson et al. 2010; Padial et al. 2010). Here we offer a real example of natural language descriptions that are annotated with semantic phenotype statements, modeled after the EQ representation referred to by Deans et al. (2012) (see also Mikó and Deans 2009; Mungall et al. 2010; Mabee et al. 2007; Patterson et al. 2010; Balhoff et al. in prep), expressed in Web Ontology Language (OWL) and therefore ready for the Semantic Web. A formal model has been developed (Balhoff et al. 2011), and its advantages and limitations are discussed by Balhoff et al. (in prep).

Our taxonomic subject is the ensign wasp genus, *Evaniscus * (Hymenoptera : Evaniidae ). Ensign wasps develop as solitary predators within cockroach egg cases (Dictyoptera : Blattodea). The family is common across the world except in polar regions, and species diversity is highest in the Neotropics (Deans 2005). There are 21 extant genera and 580 described species of Evaniidae  in the world (Deans 2005; Kawada 2012); ten genera of fossil evaniids are also known (Deans 2005; Peñalver et al. 2010). There is a paucity of prey records for Evaniidae  in general, and none is known for *Evaniscus * (Deans 2005).

*Evaniscus * Szépligeti, 1903 is a relatively small genus of New World ensign wasps with four previously known, rarely collected species (Deans and Huben 2003). The genus belongs to a New World clade that exhibits reduced wing venation, along with *Semaeomyia *, *Hyptia *, *Decevania * and *Rothevania * (Deans et al. 2006). Originally described by Szépligeti in 1903 for an unusual species from Venezuela, *Evaniscus tibialis *, the genus has not been previously revised. Only two other New World evaniid lineages, *Alobevania * and *Decevania *, have undergone revision recently (Deans and Kawada 2008, Kawada and Azevedo 2007, Kawada 2011).

Deans and Huben (2003) diagnosed *Evaniscus * by the following characters: “RS+M vein missing in the fore wing, coxae evenly-spaced, head hemispherical in lateral view, antennae 13-segmented and arising mid-height on the head, and metasoma ovoid”. In addition to the type species, three other species are currently included within *Evaniscus *: *Evaniscus marginatus * (Cameron, 1887), *Evaniscus rufithorax * Enderlein, 1905, and *Evaniscus sulcigenis * Roman, 1917.

Two hundred-fifty years of ensign wasp taxonomy has thus far yielded a corpus of species descriptions that lack utility beyond the realm of descriptive taxonomy (and even very little utility within this domain, as descriptions are usually short and lexically cryptic). For almost all Evaniidae , identification of species must be done by direct comparison with type specimens since there is a shortage of useable species descriptions or identification keys.

The three primary goals of this paper are to 1) provide diagnostic characters for the identification of *Evaniscus * species as well as a phylogeny, annotated images, and distribution records for species (i.e., a robust taxonomic revision), 2) apply new descri ptive methods, whereby annotations are composed from multiple ontologies to form semantic phenotype statements (a formal extension of methods described by Mikó and Deans 2009) and 3) assess the utility of free, online collaborative tools for use in descriptive taxonomy (an extension of methods described by Deans and Kawada 2008).

## Material and methods 

*Collaborative environment *. We used many accessible, free tools that have potential to help accelerate the publication of a manuscript. Since the authors were separated by physical distance, we used tools disseminated through the World Wide Web, such as online text editors (e.g. Google docs), Google draw, and Flickr (http://flickr.com  ) that allowed for immediate and efficient communication. Matrix-based species descriptions were generated from mx (Yoder et al. 2006), a free, open-source software program for systematic biologists, which is designed to store various specimen metadata and to export the data as free text (in the format of “Character: Character state(s)”) and as input files that can be used in other applications.

*Characters *. Characters were described in natural language and then annotated with formalized entity-quality (EQ) statements (Washington et al. 2009), where an anatomical structure is an entity and a phenotype descriptor represents a quality. EQ statements were composed using the following ontologies available through the Open Biomedical Ontologies Foundry: Hymenoptera  Anatomy Ontology (HAO, Yoder et al. 2010) version *<*http://purl.obolibrary.org/obo/hao/2012-05-03/hao.owl *>*, Phenotypic Quality Ontology (PATO, Mungall et al. 2010) version < http://purl.obolibrary.org/obo/pato/2012-05-09/pato.owl> , Relation Ontology (RO) (07/11/2012, 8:58 <http://purl.obolibrary.org/obo/ro.owl> ) and Spatial Ontology (BSPO) (05/18/2012, 9:04 <http://purl.obolibrary.org/obo/bspo.owl> ). Wing vein terminology is included from Deans and Huben (2003). Fifty-six morphological characters, with 137 character states (Appendix I), were scored for all *Evaniscus * species and outgroups treated in this study. All characters and character states are available in Appendix A.

*Measurements *. Mesosoma length is measured in lateral view from the anterior-most point of the pronotum to the posterior-most point of the metapectal-propodeal complex. All measurements were made with an ocular micrometer, installed inside an Olympus SZX16 Research Stereo Microscope.

*Semantic phenotype development *. 1) All phenotype data were captured in mx as a character matrix; 2) Descriptive matrix elements and mx-generated specimen identifiers were exported to OWL (Web Ontology Language, http://www.w3.org/TR/owl2-overview/ ); 3) OWL-formatted data from mx were loaded along with HAO, PATO, RO, and BSPO into Protégé 4.1 (http://protege.stanford.edu/ ); 4) Semantic phenotype annotations were manually added to character states within Protégé as OWL class expressions using the built-in Manchester syntax (http://www.w3.org/TR/owl2-manchester-syntax/ ) editor. All phenotype statements in Manchester syntax are available in Appendix B.

*Phylogenetics *. Outgroups from four different genera were chosen from the closest known relatives based on estimated evaniid relationships using 16S and 28S ribosomal RNA (rRNA) data in MrBayes (Deans et al. 2006) and morphological similarity to *Evaniscus * (Deans and Huben 2003), including a more distantly related species, *Alobevania gattiae * Kawada and Deans 2008). To discover new characters of phylogenetic importance, we examined as many individuals of each species as possible and extracted homologous characters across species. A total of 31 parsimony-informative morphological characters were analyzed in this study. A parsimony analysis was performed with an exhaustive search in PAUP* version 4, beta 10 (Swofford 2002). The root was placed at *Alobevania gattiae *. Jackknife and bootstrap values were calculated using default settings with 1000 pseudoreplications.

*Data management *. Morphological characters, taxonomic concepts, descriptive language, electronic keys, and georeferenced collecting events were maintained in mx (Yoder et al. 2006). Over twenty researchers currently contribute to the development of the Hymenoptera  Anatomy Ontology (Yoder et. al 2010). Phylogenetic datasets, trees and associated metadata, such as specimen information and matrices, were exported from mx as NeXML and are deposited into TreeBASE. Semantic, marked-up phenotype annotations expressed in OWL are deposited in the Dryad Data repository. Mx-generated species pages are provided to the Encyclopedia of Life via XML exports.

*Distribution map *. Google Maps **^®^** is used to produce distribution maps for each species. Collecting locality data are available on species pages at the Evanioidea  Online (http://evanioidea.info/ ) descriptive web pages and are also shared with EOL.

*Images *. Specimens were examined using an Olympus SZX16 Research Stereo Microscope (at NCSU) and Leica MZ12.5 (at MZSP). Images for figures were obtained using the Passport Storm Portable Imaging System by Visionary Digital and combined with Combine ZP**^©^** (Hadley 2009) or a Leica M205C magnifying glass attached to a Leica DFC 295 video camera with images combined using Leica LAS (Leica Application Suite V3.6.0) Microsystems by Leica (Switzerland) Limited. All images were cropped and brightness and contrast were adjusted in Adobe Photoshop **^®^** CS4 when necessary. Images included within this study are available at Morphbank (http://morphbank.net  ).

*Material examined *. Specimens (Appendix C) were borrowed from museums (see Acknowledgments). Nine specimens of *Evaniscus rufithorax * and four specimens of *Evaniscus marginatus * (including the holotype for *Evaniscus marginatus * and three syntypes of *Evaniscus rufithorax *), and two additional specimens of *Evaniscus tibialis * were observed and imaged at the Natural History Museum in London, UK and Museum für Naturkunde, Berlin, Germany, but were not assigned NCSU barcode numbers.

## Data resources 

The data underpinning the analyses reported in this paper are deposited in the Dryad Data Repository at doi: 10.5061/dryad.2jd88 and at TreeBASE (http://purl.org/phylo/treebase/phylows/study/TB2:S13316 ).

## Results 

### Taxonomy 

#### Evaniscus

Szépligeti, 1903

http://species-id.net/wiki/Evaniscus

Evaniscus : Szépligeti, 1903 (original description)
Evaniscus : Szépligeti, G. 1903: 378
Pseudevania : Bradley, J. C. 1905: 63–64 (misspelling)

##### Diagnosis.

Members of the genus *Evaniscus * are distinguished from other Evaniidae  by a combination of the following character states: Fore wing RS+M vein presence: absent; mesosternum length vs metasternum length: ventral margin of mesosternum length equal to ventral margin of metapectus length; head shape: hemispherical in lateral view; flagellomere number: 13; metasoma shape in lateral view: ovoid; mandibular teeth number: 2; metanotum sculpture: scrobiculate; mesoscutellum sculpture: foveate; metapectal-propodeal complex sculpture: areolate; vertex sculpture: foveate; carinae on gena presence: present; notauli presence: present; parapsidal signum presence: present; subantennal carinae presence: present; preorbital carinae presence: present.

##### Description.

*Head *. Mandibular teeth number: 2. Subantennal carina presence: present. Preorbital carina presence: present. Carinae  on gena presence: present. Vertex sculpture: foveate. Radicle sculpture: punctate.

*Mesosoma *. Mesosternum length vs. metasternum length: ventral margin of mesosternum length equal to ventral margin of metapectus length. Metanotum sculpture: scrobiculate. Mesoscutellum sculpture: foveate. Metapectal propodeal complex surface feature shape: areolate. Notaulus presence: present. Parapsidal signum presence: present.

*Legs *. Metatibial spur length: inner metatibial spur greater than 1.3× as long as outer spur. Spines on posterior area of metatibia presence: present.

*Wings *. Fore wing length: extending beyond posterior margin of metasoma. Fore wing cell count: 6 cells. Fore wing RS+M vein presence: absent. Hind wing jugal region presence: present.

#### Evaniscus lansdownei

Mullins sp. n.

urn:lsid:zoobank.org:act:1EA35281-4E0D-4317-A533-39A905883629

http://species-id.net/wiki/Evaniscus_lansdownei

Figures 1–6

##### Etymology.

This species is named in honor of four sixth-grade students (Donyae Johnson, Monique McRae, Breeanna Berrios and Iyanna Reeves) at Lansdowne Middle School, Baltimore, MD, for winning the Hexapod Haiku challenge at North Carolina State University in 2011.

##### Diagnosis.

*Evaniscus lansdownei * is easily recognized by two unique characters: fore wing vein color: yellow; setae on proximal region of fore wing color: yellow.

##### Description.

*Head *. Head color: yellow. Mandible color vs clypeus color: mandible color same as clypeus color. Subantennal carina length: extending dorsally of medial margin of lower face. Preorbital carina length: extending dorsally to ventral margin of the antennal foramen. Upper face sculpture: punctate and foveate. Malar space length vs. half compound eye height (male): shorter than half compound eye height. Ocellar ocular line length vs. lateral ocellus diameter: as long or longer than lateral ocellus diameter. Posterior ocellar length vs. lateral ocellus diameter: 1.5× as long as the diameter of the lateral ocellus. Ventral region of occipital carina curvature in lateral view: straight. Radicle color: yellow. Scape color: yellow. Scape length vs compound eye height: scape shorter than half compound eye height.

*Mesosoma *. Mesosoma length: 3.5–3.5 mm (n=1). Antero-dorsal region of mesosoma color: yellow. Postero-ventral region of mesosoma color: black. Median notch of transverse pronotal carina presence: present. Transverse pronotal carina length: long, extending postero-laterally of epomia. Pronotal collar sculpture: foveate. Patch that is part of dorsal region of lateral pronotal area texture: smooth. Pronotal lobe carina presence: present. Mesonotum color: red. Mesoscutum shape: as long as wide (length of mesoscutum > width of mesoscutum). Antero-admedian line length vs. lateral ocellus diameter: equal to lateral ocellus diameter. Parapsidal signum conspicuousness: inconspicuous. Foveae on notaulus presence: present. Distance between depressions vs. diameter of depressions on internotaular area: greater than the diameter of one depression. Mesofemoral depression sculpture: smooth. Mesofemoral depression pilosity presence: absent. Ventral area of the mesopectus sculpture: smooth. Medial region of transmetapectal carina presence: absent. Area dorsal of transmetapectal carina sculpture: areolate. Posterior propodeal projection shape in lateral view: not raised. Posterior region of plica presence: present. Dorsal area of the metapectal-propodeal complex sculpture: foveate. Posterior margin of the metapectal-propodeal complex ventrally of the propodeal foramen curvature in lateral view: curved. Mesosoma color: black posteroventrally, yellow anterodorsally.

*Legs *. Fore leg color: yellow. Mid leg color: yellow. Hind leg color: black. Metafemur length vs. metatibia length: metafemur equal to or shorter than metatibia. Metabasitarsus length vs metatibia length: metabasitarsus 1.2× to 1.4× as short as metatibia.

*Wings *. Fore wing vein color: yellow. Setae on proximal region of fore wing color: yellow.

*Metasoma *. Metasoma color: black. Dorsal region of petiole sculpture: foveate.

**Figures 1–6.**
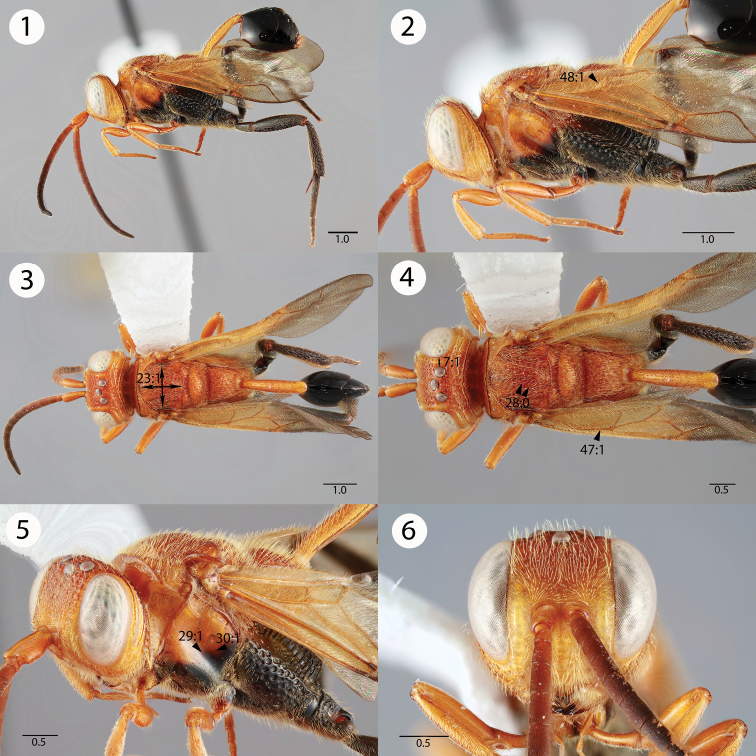
Brightfield images of *Evaniscus lansdownei * Mullins sp. n. **1, 2** Lateral habitus **3, 4** Dorsal habitus **5** Anterior oblique **6** Anterior face.

##### Material examined.

HOLOTYPE male: COLOMBIA: Mata Mata Sta., Sweep, 8–12..2000, M. Sharkey, NCSU 33809 (deposited in NCSU). Paratypes (1 male). BRAZIL: NCSU 67242 (INPA).

#### Evaniscus marginatus

(Cameron, 1887)

http://species-id.net/wiki/Evaniscus_marginatus

Figures 19–24

Evania marginatus : Cameron, 1887 (original description) holotype female, deposited at BMNH, labels: “Guatemala, Capetillo (Champion)”, mx_id: 479; holotype female, deposited at BMNH, labels: “Guatemala, Capetillo (Champion)”, mx_id: 15348
Evania marginata : Cameron, P. 1887: 430 (genus transfer)
Pseudevania marginata : Kieffer, J. J. 1903: 111 (genus transfer, misspelling)
Zeuxevania marginata : Kieffer, J. J. 1904: 395 (genus transfer)
Evaniscus marginatus : Bradley, J. C. 1908: 180

##### Diagnosis.

*Evaniscus marginatus * is recognized by the combination of the following character states: subantennal carina length: extending dorsally of medial margin of lower face; pronotal lobe carina presence: absent; mesofemoral depression sculpture: foveate; mesofemoral depression pilosity presence: present.

##### Description.

*Head *. Head color: dorsal half of upper face and vertex color black; ventral half of upper face and lower face color red or yellow. Mandible color vs clypeus color: mandible color different than clypeus color; mandible color same as clypeus color. Subantennal carina length: extending dorsally of medial margin of lower face. Preorbital carina length: extending dorsally to the ventral margin of the anterior ocellus. Upper face sculpture: foveate. Malar space length vs. half compound eye height (male): shorter than half compound eye height. Ocellar ocular line length vs. lateral ocellus diameter: shorter than lateral ocellus diameter. Posterior ocellar length vs. lateral ocellus diameter: 1.5× as long as the diameter of the lateral ocellus. Ventral region of occipital carina curvature in lateral view: straight. Ventral region of the postoccipital carina shape: not raised. Radicle color: yellow; orange. Scape color: yellow; orange. Scape length vs compound eye height: scape shorter than half compound eye height. Female flagellomere 1-8 shape: distinctly wider than long (length of flagellomere < width of flagellomere). Shape of occiput: as high as wide.

*Mesosoma *. Mesosoma length: 2.75–2.75 mm (n=4). Antero-dorsal region of mesosoma color: black. Postero-ventral region of mesosoma color: black. Median notch of transverse pronotal carina presence: present. Transverse pronotal carina length: long, extending postero-laterally of epomia. Pronotal collar sculpture: scrobiculate and foveate. Patch that is part of dorsal region of lateral pronotal area texture: smooth. Pronotal lobe carina presence: absent. Mesonotum color: black. Mesoscutum shape: as long as wide (length of mesoscutum > width of mesoscutum). Antero-admedian line length vs. lateral ocellus diameter: equal to lateral ocellus diameter. Parapsidal signum conspicuousness: inconspicuous. Foveae on notaulus presence: absent. Distance between depressions vs. diameter of depressions on internotaular area: greater than the diameter of one depression. Mesofemoral depression sculpture: foveate. Mesofemoral depression pilosity presence: present. Ventral area of the mesopectus sculpture: foveate. Medial region of transmetapectal carina presence: absent. Area dorsal of transmetapectal carina sculpture: smooth. Posterior propodeal projection shape in lateral view: not raised. Posterior region of plica presence: absent. Dorsal area of the metapectal-propodeal complex sculpture: foveate. Posterior margin of the metapectal-propodeal complex ventrally of the propodeal foramen curvature in lateral view: curved. Mesosoma color: black.

*Legs *. Fore leg color: yellow; red. Mid leg color: yellow; red. Hind leg color: black. Metafemur length vs. metatibia length: metafemur equal to or shorter than metatibia. Metabasitarsus length vs metatibia length: metabasitarsus 1.4× to 1.6× as short as metatibia.

*Wings *. Fore wing vein color: black. Setae on proximal region of fore wing color: black.

*Metasoma *. Metasoma color: black. Dorsal region of petiole sculpture: foveate.

##### Material examined.

Holotype female: GUATEMALA: (deposited in BMNH). Other material (9 females, 2 males): BRAZIL: 2 females. NCSU 67240-67241 (MZSP). COSTA RICA: 5 females, 2 males. NCSU 9892 (AEIC); NCSU 9893 (UCDC); mx_id 15343-15346 (BMNH); Deans Lab Legacy Identifiers DERV052 (INBC). ECUADOR: 1 female. NCSU 41748 (USNM). MEXICO: 1 female. NCSU 9894 (TAMU).

#### Evaniscus rafaeli

Kawada sp. n.

urn:lsid:zoobank.org:act:C580F20D-5107-4715-9284-BB21F629004E

http://species-id.net/wiki/Evaniscus_rafaeli

Figures 7–12 31 –32

##### Etymology.

The specific epithet honors José Albertino Rafael, a great collector in the Amazon forest and an entomologist at INPA.

##### Diagnosis.

This species shares the following character states with *Evaniscus tibialis *: posterior margin of the metapectal-propodeal complex ventrally of the propodeal foramen curvature in lateral view: straight; scape length vs compound eye height: scape longer than half compound eye height; mesoscutum shape: wider than long (length of mesoscutum < width of mesoscutum); dorsal region of petiole sculpture: wrinkled. The following character states are present in *Evaniscus rafaeli * but not in *Evaniscus tibialis *: ventral region of occipital carina curvature in lateral view: straight; median notch of transverse pronotal carina presence: present; transverse pronotal carina length: long, extending postero-laterally of epomia; parapsidal signum conspicuousness: inconspicuous.

##### Description.

*Head *. Head color: orange. Mandible color vs clypeus color: mandible color same as clypeus color. Subantennal carina length: extending dorsally of medial margin of lower face. Preorbital carina length: extending dorsally to the ventral margin of the anterior ocellus. Upper face sculpture: foveate. Malar space length vs. half compound eye height (male): shorter than half compound eye height. Ocellar ocular line length vs. lateral ocellus diameter: as long or longer than lateral ocellus diameter. Posterior ocellar length vs. lateral ocellus diameter: 1.5× as long as the diameter of the lateral ocellus. Ventral region of occipital carina curvature in lateral view: straight. Ventral region of the postoccipital carina shape: raised. Radicle color: red. Scape color: red. Scape length vs compound eye height: scape longer than half compound eye height. Female flagellomere 1-8 shape: distinctly wider than long (length of flagellomere < width of flagellomere). Shape of occiput: as high as wide.

*Mesosoma *. Mesosoma length: 2.0–2.0 mm (n=4). Antero-dorsal region of mesosoma color: red. Postero-ventral region of mesosoma color: red. Median notch of transverse pronotal carina presence: present. Transverse pronotal carina length: long, extending postero-laterally of epomia. Pronotal collar sculpture: scrobiculate and foveate. Patch that is part of dorsal region of lateral pronotal area texture: smooth. Pronotal lobe carina presence: present. Mesonotum color: red. Mesoscutum shape: wider than long (length of mesoscutum < width of mesoscutum). Antero-admedian line length vs. lateral ocellus diameter: equal to lateral ocellus diameter. Parapsidal signum conspicuousness: inconspicuous. Foveae on notaulus presence: present. Distance between depressions vs. diameter of depressions on internotaular area: less than the diameter of one depression. Mesofemoral depression sculpture: smooth. Mesofemoral depression pilosity presence: absent. Ventral area of the mesopectus sculpture: smooth. Medial region of transmetapectal carina presence: absent. Area dorsal of transmetapectal carina sculpture: areolate. Posterior propodeal projection shape in lateral view: not raised. Posterior region of plica presence: absent. Dorsal area of the metapectal-propodeal complex sculpture: foveate. Posterior margin of the metapectal-propodeal complex ventrally of the propodeal foramen curvature in lateral view: straight. Mesosoma color: red.

*Legs *. Fore leg color: red. Mid leg color: red. Hind leg color: red-black. Metafemur length vs. metatibia length: metafemur longer than metatibia. Metabasitarsus length vs metatibia length: metabasitarsus 1.2× to 1.4× as short as metatibia.

*Wings *. Fore wing vein color: black. Setae on proximal region of fore wing color: black.

*Metasoma *. Metasoma color: black. Dorsal region of petiole sculpture: wrinkled.

**Figures 7–12.**
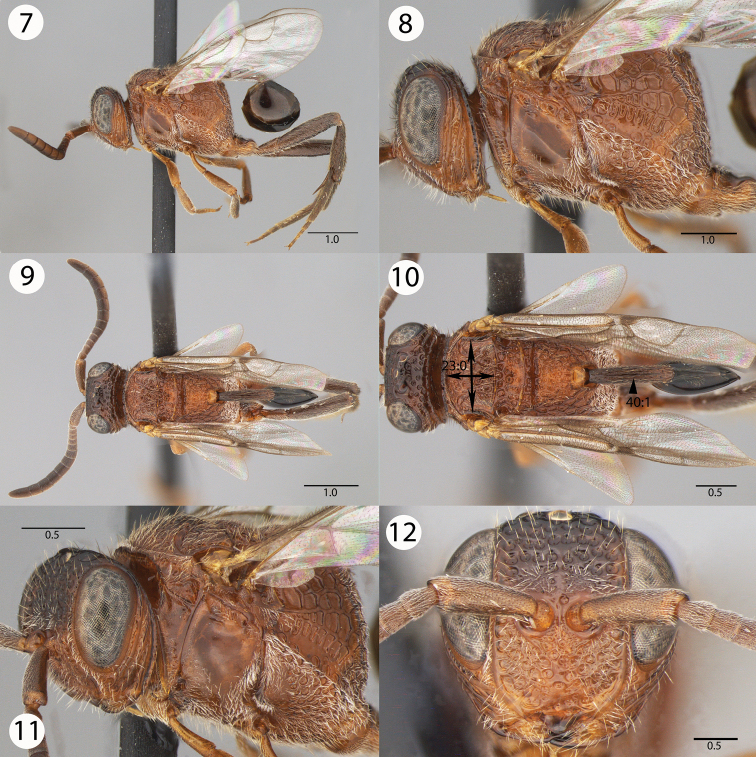
Brightfield images of *Evaniscus rafaeli * Kawada sp. n. **7, 8** Lateral habitus **9, 10** Dorsal habitus **11** Anterior oblique **12** Anterior face.

**Figures 13–18.**
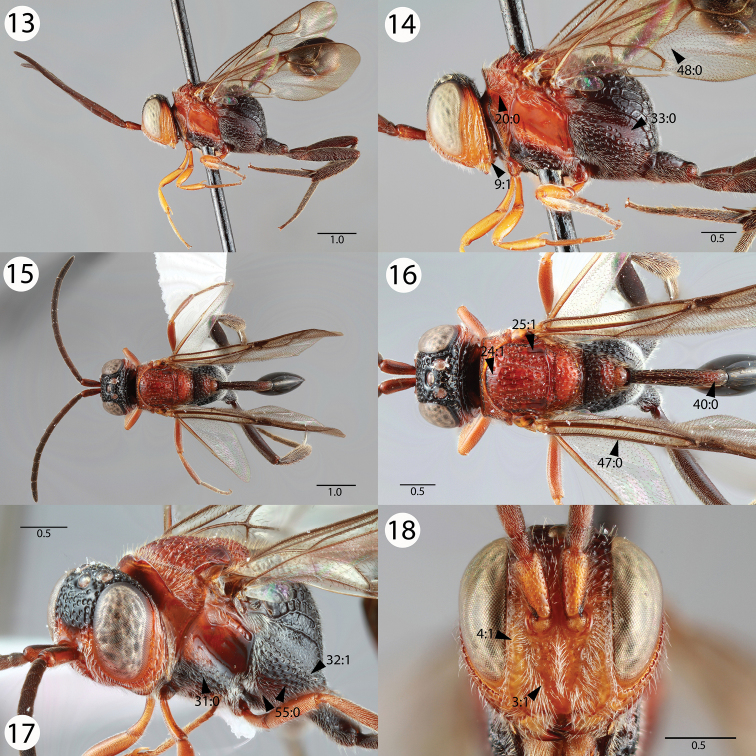
Brightfield images of *Evaniscus rufithorax * Enderlein. **13, 14** Lateral habitus **15, 16** Dorsal habitus **17** Anterior oblique **18** Anterior face.

**Figures 19–24.**
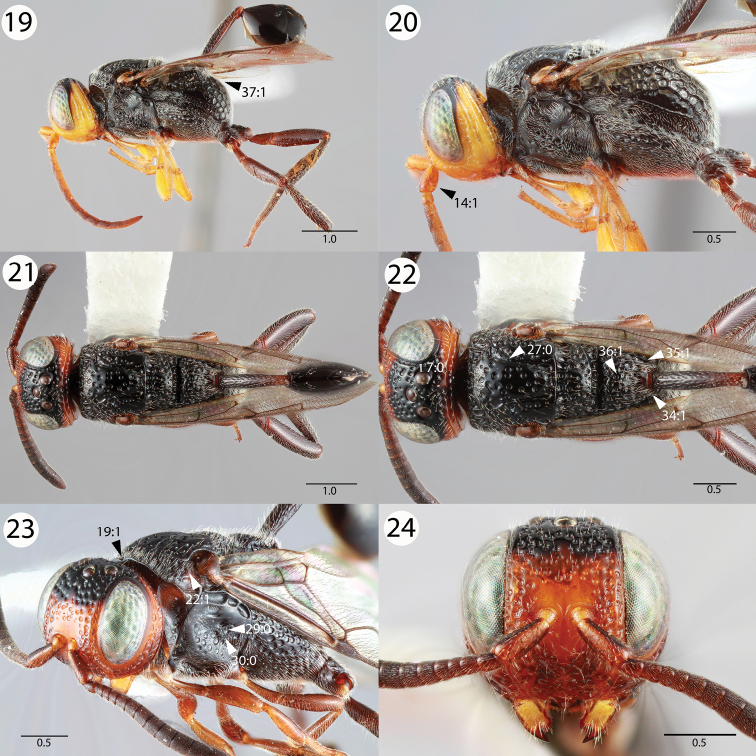
Brightfield images of *Evaniscus marginatus * Cameron. **19, 20** Lateral habitus **21, 22** Dorsal habitus **23** Anterior oblique **24** Anterior face.

##### Material examined.

HOLOTYPE female: BRAZIL: Manaus Reserva [Florestal Adolpho] Ducke, 26 Km NE Manaus, Arm. suspensa 21m, 22.1988, JA Rafael, NCSU 0067245 (deposited in INPA). Paratypes (3 females): BRAZIL: 3 females. NCSU 67243-67244, 67246 (INPA).

#### Evaniscus rufithorax

Enderlein, 1905

http://species-id.net/wiki/Evaniscus_rufithorax

Figures 13–18

Evaniscus rufithorax : Enderlein, 1905 (original description) male, deposited at ZMPA, labels: “Bolivien. Mapiri and Peru: Pachita{Pachitea}-Fluß”, mx_id: 480; syntype male, deposited at ZMHB, labels: “Bolivia Mapiri Staudinger, V.”, mx_id: 15349; syntype male, deposited at ZMHB, labels: “Bolivia Mapiri Staudinger, V.”, mx_id: 15350; syntype male, deposited at ZMHB, labels: “Peru Pachitea-Fluss Staudinger, V.”, mx_id: 15351
Evaniscus rufithorax : Enderlein, G. 1905: 711

##### Diagnosis.

*Evaniscus rufithorax * is the most commonly collected species of in the genus. This species differs from other *Evansicus * by a combination of the following character states: pronotal lobe carina presence: present; subantennal carina length: extending ventrally of medial margin of lower face.

##### Description.

*Head *. Head color: dorsal half of upper face and vertex color black; ventral half of upper face and lower face color red or yellow; orange. Mandible color vs clypeus color: mandible color same as clypeus color. Subantennal carina length: extending ventrally of medial margin of lower face. Preorbital carina length: extending dorsally to ventral margin of the antennal foramen. Upper face sculpture: foveate. Malar space length vs. half compound eye height (male): shorter than half compound eye height. Ocellar ocular line length vs. lateral ocellus diameter: shorter than lateral ocellus diameter. Posterior ocellar length vs. lateral ocellus diameter: 1.5x as long as the diameter of the lateral ocellus. Ventral region of occipital carina curvature in lateral view: straight. Ventral region of the postoccipital carina shape: not raised. Radicle color: yellow; orange. Scape color: yellow; orange. Scape length vs compound eye height: scape shorter than half compound eye height. Female flagellomere 1-8 shape: distinctly longer than wide (length of flagellomere > width of flagellomere). Shape of occiput: as high as wide.

*Mesosoma *. Mesosoma length: 2.25–2.85 mm (n=29). Antero-dorsal region of mesosoma color: red. Postero-ventral region of mesosoma color: black; red. Median notch of transverse pronotal carina presence: present. Transverse pronotal carina length: long, extending postero-laterally of epomia. Pronotal collar sculpture: foveate. Patch that is part of dorsal region of lateral pronotal area texture: smooth. Pronotal lobe carina presence: present. Mesonotum color: red; black. Mesoscutum shape: as long as wide (length of mesoscutum > width of mesoscutum). Antero-admedian line length vs. lateral ocellus diameter: equal to lateral ocellus diameter. Parapsidal signum conspicuousness: inconspicuous. Foveae on notaulus presence: present. Distance between depressions vs. diameter of depressions on internotaular area: greater than the diameter of one depression. Mesofemoral depression sculpture: smooth. Mesofemoral depression pilosity presence: absent. Ventral area of the mesopectus sculpture: foveate. Medial region of transmetapectal carina presence: absent. Area dorsal of transmetapectal carina sculpture: smooth. Posterior propodeal projection shape in lateral view: not raised. Posterior region of plica presence: absent. Dorsal area of the metapectal-propodeal complex sculpture: foveate. Posterior margin of the metapectal-propodeal complex ventrally of the propodeal foramen curvature in lateral view: curved. Mesosoma color: black posteroventrally, red anterodorsally.

*Legs *. Fore leg color: yellow; red. Mid leg color: yellow; red. Hind leg color: red-black. Metafemur length vs. metatibia length: metafemur equal to or shorter than metatibia. Metabasitarsus length vs metatibia length: metabasitarsus 1.2× to 1.4× as short as metatibia.

*Wings *. Fore wing vein color: black. Setae on proximal region of fore wing color: black.

*Metasoma *. Metasoma color: black. Dorsal region of petiole sculpture: foveate.

##### Material examined.

Lectotype male, current designation: PERU: 1 male. mx_id 15351 (ZMHB). Paralectotypes: 3 males, current designation: BOLIVIA: 3 males. mx_id 15349-15350 (ZMHB); mx_id 23037 (ZMPA). Other material (21 females, 77 males, 1 sex unknown): BRAZIL: 8 females, 31 males, 1 sex unknown. NCSU 2395-2400, 6967-6968, 41750 (AEIC); NCSU 67257-67273, 67279 (INPA); NCSU 9888-9889 (UCDC); NCSU 67280-67286 (MPEG); NCSU 67287-67290 (MZSP). COLOMBIA: 1 female, 4 males. NCSU 33582, 36699, 41741-41742 (NCSU); mx 2840 (unknown). ECUADOR: 9 females, 23 males. NCSU 6969-6974, 6976-6977, 6986-7000 (AEIC); NCSU 6979-6985 (UCDC); NCSU 6975 (INHS); NCSU 6978 (USNM). GUYANA: 1 male. mx_id 15338 (BMNH). PERU: 3 females, 15 males. NCSU 18398-18399 (MZLU); NCSU 67277-67278 (MIUP); NCSU 9886-9887, 9890-9891 (AEIC); mx_id 15340-15342 (BMNH); NCSU 2391-2394 (INHS); NCSU 67274-67276 (CAS). SURINAME: 1 male. mx_id 15337 (BMNH).

##### Comments.

A lectotype was designated because syntypes were from specimens with different localities and one syntype varies in color; the male specimen chosen as lectotype from Peru is in very good condition and fits the description well. The male paralectotype from Bolivia that is at ZMPA varies in color (only) from the other type specimens and all known other material; the antero-dorsal region of mesosoma color is red in all *Evaniscus rufithorax * specimens, but is black in this type specimen.

#### Evaniscus tibialis

Szépligeti, 1903

http://species-id.net/wiki/Evaniscus_tibialis

Figures 25–30

Evaniscus tibialis : Szépligeti, 1903 (original description) holotype female, deposited at HNHM, labels: “Venezuela: Merida”, mx_id: 482; holotype female, deposited at HNHM, labels: “Merida Venezuela 539 135 Evaniscus tibialis Szepl. id.nr.015742 HNHM Hym. Coll. “, mx_id: 15132
Evaniscus tibialis : Szépligeti, G. 1903: 378

##### Diagnosis.

*Evaniscus tibialis * is the largest species of the genus, is the only species that ever has an entirely black body, and can be distinguished from other species by the combination of the following characters: ocellar ocular line length vs lateral ocellus diameter: as long or longer than lateral ocellus diameter; transverse pronotal carina length: short, not extending postero-laterally of epomia; parapsidal signum conspicuousness: conspicuous; posterior propodeal projection shape in lateral view: raised.

##### Description.

*Head *. Head color: black; dorsal half of upper face and vertex color black; ventral half of upper face and lower face color red. Mandible color vs clypeus color: mandible color same as clypeus color. Subantennal carina length: extending dorsally of medial margin of lower face. Preorbital carina length: extending dorsally to the ventral margin of the anterior ocellus. Upper face sculpture: foveate. Malar space length vs half compound eye height (male): as long as or longer than half compound eye height. Ocellar ocular line length vs. lateral ocellus diameter: as long or longer than lateral ocellus diameter. Posterior ocellar length vs. lateral ocellus diameter: 1.5× as long as the diameter of the lateral ocellus. Ventral region of occipital carina curvature in lateral view: curved. Ventral region of the postoccipital carina shape: raised. Radicle color: black; red. Scape color: black; red. Scape length vs compound eye height: scape longer than half compound eye height. Female flagellomere 1-8 shape: distinctly longer than wide (length of flagellomere > width of flagellomere). Shape of occiput: higher than wide.

*Mesosoma *. Mesosoma length: 3.5mm (n=4). Antero-dorsal region of mesosoma color: black. Postero-ventral region of mesosoma color: black. Median notch of transverse pronotal carina presence: absent. Transverse pronotal carina length: short, not extending postero-laterally of epomia. Pronotal collar sculpture: foveate. Patch that is part of dorsal region of lateral pronotal area texture: wrinkled. Pronotal lobe carina presence: present. Mesonotum color: black. Mesoscutum shape: wider  than long (length of mesoscutum < width of mesoscutum). Antero-admedian line length vs. lateral ocellus diameter: greater than lateral ocellus diameter. Parapsidal signum conspicuousness: conspicuous. Foveae on notaulus presence: present. Distance between depressions vs. diameter of depressions on internotaular area: less than the diameter of one depression. Mesofemoral depression sculpture: smooth. Mesofemoral depression pilosity presence: absent. Ventral area of the mesopectus sculpture: smooth. Medial region of transmetapectal carina presence: present. Area dorsal of transmetapectal carina sculpture: areolate. Posterior propodeal projection shape in lateral view: raised. Posterior region of plica presence: present. Dorsal area of the metapectal-propodeal complex sculpture: areolate. Posterior margin of the metapectal-propodeal complex ventrally of the propodeal foramen curvature in lateral view: straight. Mesosoma color: black.

*Legs *. Fore leg color: red. Mid leg color: red. Hind leg color: black. Metafemur length vs. metatibia length: metafemur longer than metatibia. Metabasitarsus length vs metatibia length: equal.

*Wings *. Fore wing vein color: black. Setae on proximal region of fore wing color: black.

*Metasoma *. Metasoma color: black. Dorsal region of petiole sculpture: wrinkled.

**Figures 25–30.**
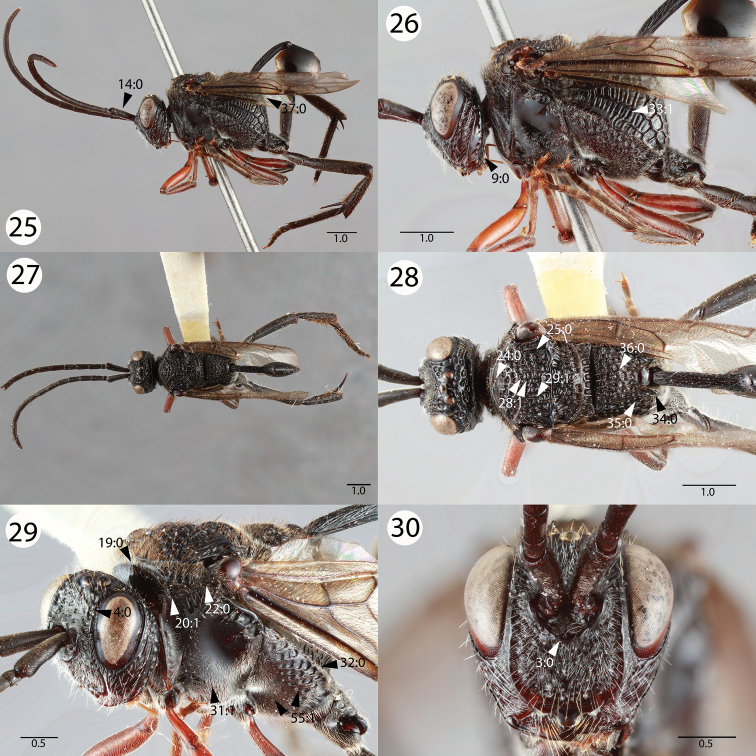
Brightfield images of *Evaniscus tibialis * Szépligeti. **25, 26** Lateral habitus **27, 28** Dorsal habitus **29** Anterior oblique **30** Anterior face.

##### Material examined.

Holotype female: VENEZUELA: HNHM Hym Coll., (deposited in HNHM). Other material (5 females, 11 males): BRAZIL: 4 females, 6 males. NCSU 67247-67255 (INPA); NCSU 67256 (MPEG). GUYANA: 2 males. mx_id 15339, 15347 (BMNH). TRINIDAD AND TOBAGO: 1 female, 3 males. NCSU 9896, 41745-41747 (USNM).

### Key to the species of *Evaniscus * for the New World 

**Table:** 

1	Ocellar ocular line length vs. lateral ocellus diameter: shorter than lateral ocellus diameter; Transverse pronotal carina length: long, extending postero-laterally of the epomia (Fig. 14, 20:0); parapsidal signum conspicuousness: inconspicuous (Fig. 16, 25:1); posterior propodeal projection shape in lateral view: not raised (Fig. 22, 34:1)	2
–	Ocellar ocular line length vs lateral ocellus diameter: as long or longer than lateral ocellus diameter; transverse pronotal carina length: short, not extending postero-laterally of epomia (Fig. 29, 20:1); parapsidal signum conspicuousness: conspicuous (Fig. 28, 25:0); posterior propodeal projection shape in lateral view: raised (Fig. 28, 34:0)	*Evaniscus tibialis * Szépligeti
2	Mesoscutum as long as wide (length of mesoscutum > width of mesoscutum) (Fig. 3, 23:1); petiole sculpture: foveate (Fig. 16, 40:0); posterior margin of the metapectal-propodeal complex ventrally of the propodeal foramen curvature in lateral view: curved (Fig. 19, 37:1)	3
–	Mesoscutum wider than long (length of mesoscutum < width of mesoscutum)(Fig. 10, 23:0); petiole sculpture: wrinkled (Fig. 10, 40:1); posterior margin of the metapectal-propodeal complex ventrally of the propodeal foramen curvature in lateral view: straight (Fig. 25, 37:0)	*Evaniscus rafaeli * Kawada, sp. n.
3	Fore wing vein color: black (Fig. 16, 47:0); setae on proximal region of fore wing color: black (Fig. 14, 48:0)	4
–	Fore wing vein color: yellow (Fig. 4, 47:1); setae on proximal region of fore wing color: yellow (Fig. 2, 48:1)	*Evaniscus lansdownei * Mullins, sp. n.
4	Pronotal lobe carina presence: present (Fig. 29, 22:0); subantennal carina length: extending ventrally of medial margin of lower face (Fig. 18, 3:1)	*Evaniscus rufithorax * Enderlein
–	Pronotal lobe carina presence: absent (Fig. 23, 22:1); subantennal carina length: extending dorsally of medial margin of lower face (Fig. 30, 3:0)	*Evaniscus marginatus * Cameron

### Phylogeny 

**Figure 31.**
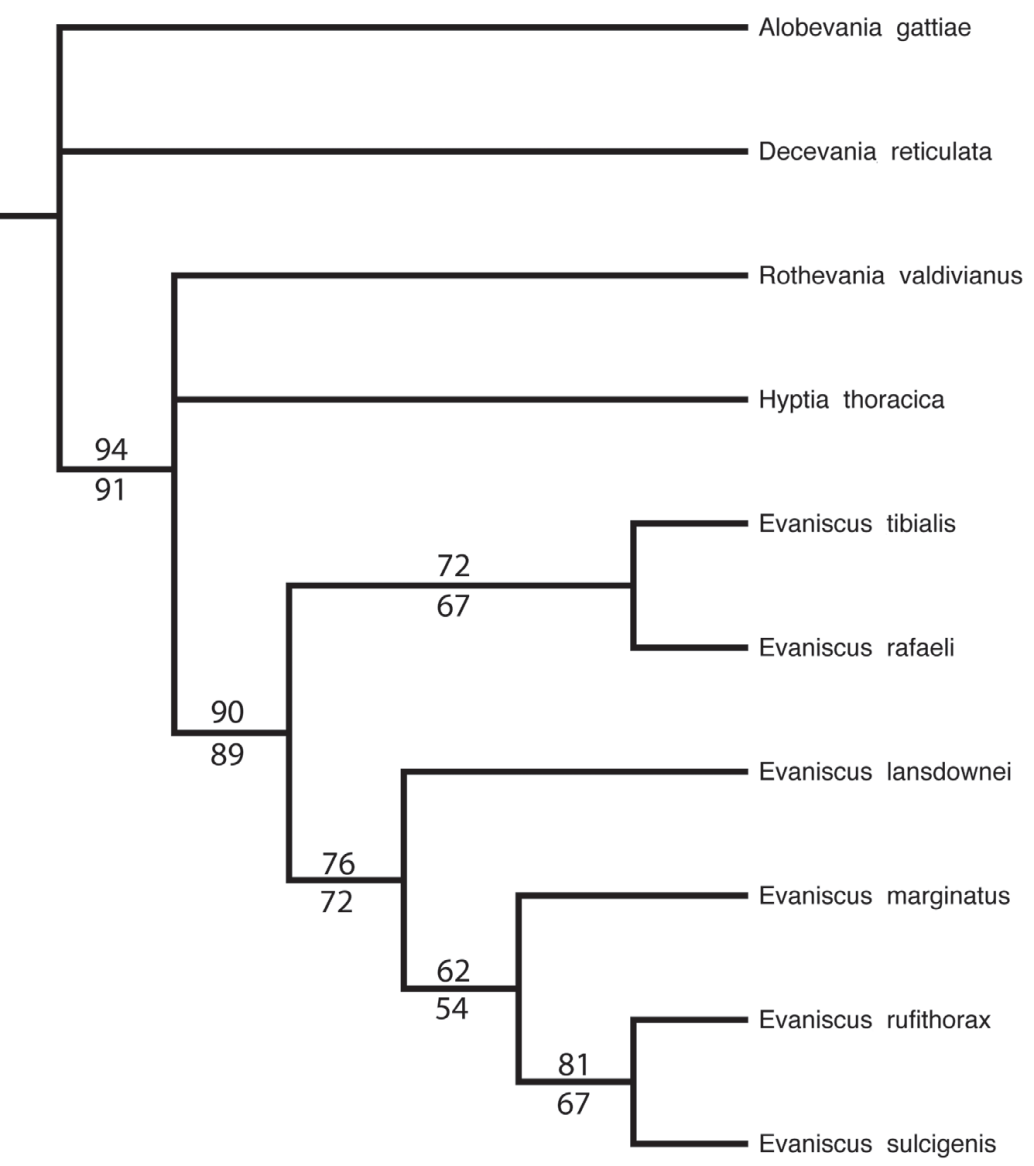
Most parsimonious tree from exhaustive search in PAUP*. Numbers above nodes show bootstrap support and numbers below nodes show jackknife support from the maximum parsimony analysis.

A single most parsimonious tree (Fig. 31) was retained from the exhaustive search in PAUP* with shortest length 70. The present morphological dataset confirms a well-supported monoplyletic *Evaniscus *.

## Discussion 

*Semantic phenotypes *. Through the descriptive process, taxonomists stand to contribute an immense body of knowledge that could be used to address a broad array of questions in many realms of biology (Deans et al. 2012). How might phenotypes be correlated to climate change? Or how might changes in phenotype correspond with the environment? Presently, queries of characters that reference a specific part of the anatomy are already possible (Deans et al. 2012). There are, however, some current limitations in the workflow of semantic phenotype construction, e.g., ontologies often do not have sufficient content, using the software to manually create the statements can be complex, and it may prove challenging for taxonomists to alter their workflow (Deans et al. 2012). Though some of the methods and tools used to build semantic phenotype annotations are in their infancy, semantic phenotypes hold the potential to unlock valuable data within taxonomic descriptions. For example, semantic phenotypes could become more meaningful when mapped across large-scale phylogenies; if we apply semantic phenotype annotations to specimens, phenotype data can be connected to evolutionary history through the organism’s phylogenetic relationships. Also, semantic phenotypes help make available millions of unambiguous data to a broad array of scientists (Deans et al. 2012).

The descriptive statements within this manuscript represent one of the first efforts in descriptive taxonomy to capture phenotypes using formalized, semantic methods. As a result of employing these new methods, a contemplative, calculated approach was taken in selecting terminology for characters and character states. The natural language descriptions in this manuscript were originally written with controlled vocabulary using terms present in ontologies that made the process of translation into semantic phenotypes relatively straightforward; for instance, a phenotype statement was written with the anatomical character followed by the descriptive character state, e.g., “dorsal area of metapectal-propodeal complex foveate”. The semantic phenotype statements resulting from these descriptions are more objective and less ambiguous than those frequently found in traditional taxonomic descriptions.

In the original description of *Evaniscus marginatus *, the mesonotum was described as “shining, bearing some large scattered punctures”. This description is vague. To make it more explicit, we changed the character name to “distance between depressions vs. diameter of depressions on internotaular area” and the states to “0: greater than (or 1: less than) the diameter of one depression”. This is more specific than the presence of some large scattered punctures on the mesonotum, but making a semantic statement from this character was not particularly intuitive (for semantic statement, see Appendix B). For the majority of characters in the descriptions presented here, the process of translation to more meaningful semantic statements was not as complicated.

Semantic phenotypes in these taxonomic descriptions were created in a logical manner by means of extracting anatomical information from an organism-specific ontology, such as the HAO, and pairing this with a quality from a general trait ontology (PATO). The natural language description persists, but a machine-readable interpretation is constructed that can be stored on the Semantic Web, where the valuable phenotypic data can easily be mined by computers and captured for use by taxonomists, biologists, or, essentially, anyone who wants to query the database of descriptions. Taxonomy that includes ontology-based descriptions, such as those presented in this manuscript, avails phenotype data to experts in all domains through bioinformatics applications.

*Geographic distribution *. In addition to the discovery of two new species, our results expand upon the geographical range of the four previously described species. Subsequently to the original descriptions, *Evaniscus tibialis * has been collected in northeast Guyana, northern Brazil, and Trinidad. The range of *Evaniscus rufithorax * has been expanded into north-central western Brazil, northeastern and southern Ecuador, and southern Colombia. *Evaniscus marginatus * was described from Guatemala, and has now been collected in Costa Rica, Mexico, Brazil and Ecuador.

The majority of described evaniid species to date are from tropical regions (Deans and Huben 2003), which is consistent with the primarily tropical and subtropical distribution of cockroaches (Vélez 2008). In Colombia alone, there are 133 species of cockroaches present, 10 of which are found in Amazonas and five of which are non-blaberids (Vélez 2008). Since the holotype of *Evaniscus lansdownei * was collected in Amazonas, one of these cockroach species could potentially represent the host of *Evaniscus lansdownei *.

Interestingly, four of the *Evaniscus tibialis * specimens in this study were collected at the entrance to Tamana Caves, Trinidad. *Blaberus posticus * dominates the cockroach fauna in this area, but because this is a blaberid and retains the ootheca within the abdominal wall, as do all other species dwelling in the caves, it is highly unlikely that they could be the host of *Evaniscus tibialis * (Darlington 1970).

*Color variation *. Some insects exhibit varying levels of intraspecific polychromatism or heterochromatism. For example, Berniker and Weirauch (2012) identified species of the reduviid genus *Apiomerus * that exhibit intraspecific polychromatism; some species showed discrete color morphs while others showed only limited polychromatism in the pronotum and the corium. Some individuals of *Apiomerus californicus * collected along the Sierra Nevada mountain range in California showed an increased red pigmentation, which suggested a possible correlation between color variation and elevation. In the ichneumonid *Aphidius smithi *, adult wasps that were reared at different temperatures during the mid to late pupal stage displayed constant differences in integumental coloration, especially in the face, thorax and petiole; when wasps were reared at higher tempteratures, the face and mesothorax were orange, but when reared at lower temperatures, these parts in adults were black (Shu-Sheng and Carver 1982).

In *Evaniscus *, heterochromatism is limited to the head and mesosoma in *Evaniscus rufithorax *. The most distinct color morph of *Evaniscus rufithorax * has the dorsal half of the upper face and vertex black while the ventral half of the upper face and lower face is red or yellow. However, a few males and females have an entirely red head. A similar color pattern can be applied to the mesosoma in this species; the majority of specimens have the postero-ventral region of the mesosoma black and the antero-dorsal region red, but some males and females have an entirely red mesosoma. The same variation in head color pattern applies to some specimens of *Evaniscus tibialis *, including the holotype.

With the limited availability of specimens in this study, it is difficult to determine if there is any correlation with color variation and biogeography in *Evaniscus rufithorax *, or if rearing temperature plays a role in adult coloration. The females with entirely red heads were collected in Ecuador with the exception of one specimen collected in Brazil. This  heterochromatism is not female-limited, however, since males of the species do exhibit the same red color morph. There is geographical overlap between the red morphs and the more common specimens with the dorsal half of the upper face and vertex black and the postero-ventral region of the mesosoma black and the antero-dorsal region red. In addition to *Evaniscus *, intraspecific polychromatism has also been observed in *Hyptia thoracica * specimens collected in the Sandhills Gamelands in North Carolina, with up to 8 different color morphs all present in the same area (personal observation, PLM).

*Systematics *. In the morphological analysis, *Evansicus * was well supported as a monophyletic lineage (bootstrap support=90, jackknife support=89). *Evaniscus rafaeli * was placed as sister to *Evaniscus tibialis *, and these two share several synapomorphic character states: posterior margin of the metapectal-propodeal complex ventrally of the propodeal foramen curvature in lateral view: straight; scape length vs compound eye height: scape longer than half compound eye height; mesoscutum shape: wider than long (length of mesoscutum < width of mesoscutum); distance between depressions vs. diameter of depressions on internotaular area: less than the diameter of one depression; dorsal region of petiole sculpture: wrinkled; metafemur length vs. metatibia length: metafemur longer than metatibia. While these two species share several characteristics, the support for their monophyly is moderate (bootstrap=72, jackknife=67). Nearly 25% of parsimony informative characters were apomorphies for *Evaniscus tibialis *. Despite the morphological analysis placing *Evaniscus tibialis * as sister to *Evaniscus rafaeli *, to the naked eye this species looks distinctly different from other *Evaniscus * species, and our understanding of the placement of *Evaniscus tibialis * would benefit from future molecular analyses.

The clade of *Evaniscus * comprising *Evaniscus lansdownei *, *Evaniscus marginatus *, *Evaniscus rufithorax * and *Evaniscus sulcigenis * was moderately supported (bootstrap support=76, jackknife support=72), but all species share several derived character states: posterior margin of the metapectal-propodeal complex ventrally of the propodeal foramen curvature in lateral view: curved; scape length vs compound eye height: scape shorter than half compound eye height; mesoscutum shape: as long as wide (length of mesoscutum > width of mesoscutum); distance between depressions vs. diameter of depressions on internotaular area: greater than the diameter of one depression; dorsal region of petiole sculpture: foveate.

In addition to those published by Deans and Huben 2003, 18 characters that all *Evaniscus * species share have been documented in this manuscript. Previous to this study, very few females of *Evaniscus * had been observed, and it was thought that the ovipositor is short and completely hidden within the metasoma (Deans and Huben 2003). However, we have seen the ovipositor in several female specimens, and it extends to the tip of the metasoma; if the ovipositor was concealed when the insect was preserved, the female metasoma looks identical to that of the male.

Based on the lack of morphological variation and the results of the present analysis, we consider *Evaniscus sulcigenis * to be a junior synonym of *Evaniscus rufithorax *. Both species share all external morphological character states examined in this study, except for some variable color patterns. Further comprehensive morphological studies in addition to molecular studies are required to fully confirm this hypothesis; however, since the only available specimen of *Evaniscus sulcigenis * is the holotype specimen, these future studies may not be practical.

*Sexual dimorphism *. Evaniidae  are usually sexually dimorphic in their antennal morphology, body coloration, facial sculpturing, and/or metasomal morphology (Deans and Huben 2003; Deans and Kawada 2008). For example, females of *Decevania * have a distinctly sculptured head, flattened, small eyes, the antennal segments are enlarged progressively from the fourth flagellomere, and the posterior region of the metasoma is expanded dorso-ventrally with the ovipositor usually concealed; males usually have larger bulging eyes, antennal segments all the same diameter, and the posterior region of the metasoma constricted dorsoventrally with genitalia protracted (depending upon preservation) (Kawada and Azevedo 2007; Kawada 2011). In addition, color pattern variation in male and female specimens of many species have also been observed (personal observation). All *Evaniscus * species are identical in coloration, except for those of *Evaniscus rufithorax * (as discussed above).

Many hymenopterans have sexually dimorphic antennae (Deans and Kawada 2008; Gauld and Fitton 1987; Wharton 1980; Onagboloa et al. 2009). Antennae of most species of Evaniidae  are also sexually dimorphic (Deans and Kawada 2008; Kawada and Azevedo 2007; Kawada 2011). In *Evaniscus *, females have a ventral sensillar patch on flagellomeres 6–12 or 8–12, whereas males do not, and many females also have flagellomeres that are distinctly wider than long, where male flagellomeres are as long as wide or longer than wide. In *Evaniscus marginatus * and *Evaniscus tibialis *, for example, flagellomeres 1–8 are distinctly wider than long in the female, but not in the male. *Evaniscus rufithorax * is likely unique among *Evaniscus * species in that the antennal flagellomeres do not exhibit the flagellomere shape sexual dimorphism; however, a ventral sensillar patch on flagellomeres 6–12 is present in females but not in males. We cannot be certain this is the only species that exhibits this character state, as the male is not yet known for *Evaniscus rafaeli * and females are still unknown for *Evaniscus lansdownei * and *Evaniscus sulcigenis *.

**Figures 32–33.**
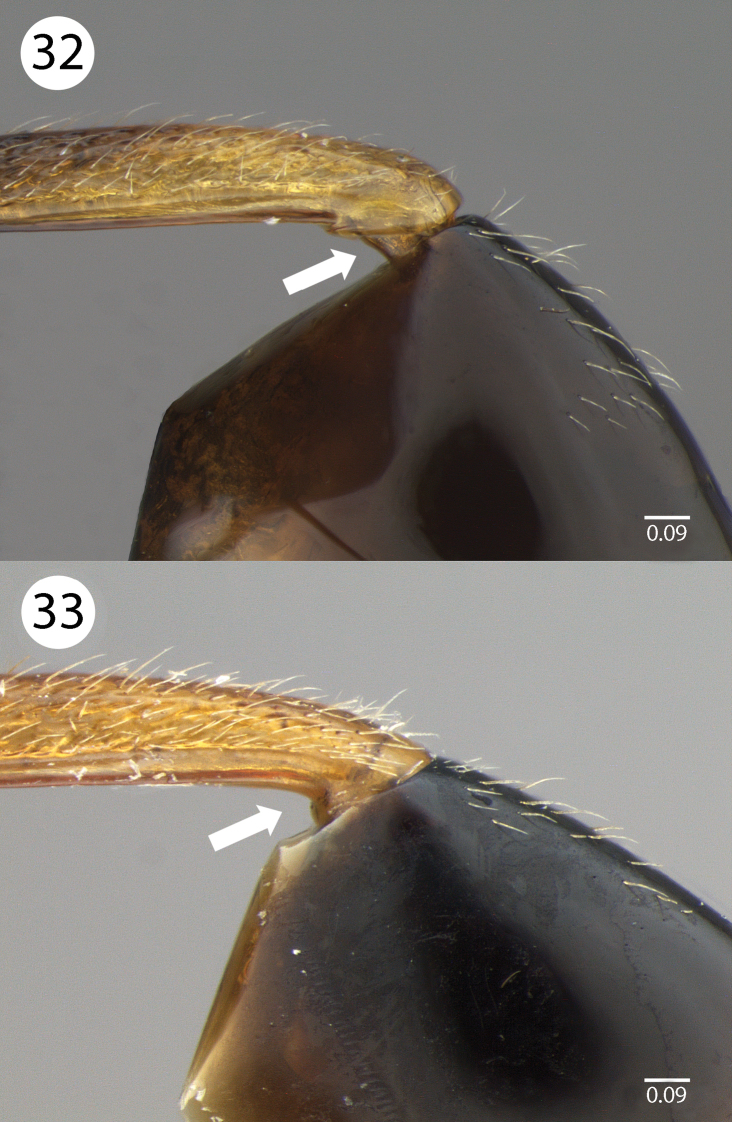
Brightfield images of *Evaniscus rufithorax *. **32** Male specimen; arrow points to visible lower tubular sclerite **33** Female specimen; arrow shows where lower tubular sclerite is not visible.

Another difference between male and female Evaniidae  specimens is the connection between the petiole and the first abdominal segment. This difference can be observed in a longitudinal section through the junction between the two sclerites in the petiole. In males, the foramen of the petiole receives the connection to the junction of the first abdominal tergite and sternite. The first abdominal tergite has a folding anterior edge along with the first abdominal sternite. These are generally divided into two sclerites: a lower tubular sclerite and another larger sclerite, which covers a large area of the first abdominal sternum (Fig. 32). In the females, the first abdominal tergite and sternite are expanded to cover the distal region of the petiole. Internally, the anterior portion of the tergite and sternite are curved to the inner wall of the petiole and connected to it by a thin membrane (Fig. 33).

## Supplementary Material

XML Treatment for Evaniscus

XML Treatment for Evaniscus lansdownei

XML Treatment for Evaniscus marginatus

XML Treatment for Evaniscus rafaeli

XML Treatment for Evaniscus rufithorax

XML Treatment for Evaniscus tibialis

## Appendix A

Character descriptions in natural language.

* marks characters and states annotated in images, and √ marks phylogenetic characters

1: Head color

(0) orange

(1) black

(2) yellow

(3) dorsal half of upper face and vertex color black

(4) ventral half of upper face and lower face color red or yellow

(5) ventral half of upper face and lower face color red

2: Mandible color vs clypeus color

(0) mandible color different than clypeus color

(1) mandible color same as clypeus color (2) black

√*3: Subantennal carina length

(0) extending dorsally of medial margin of lower face

(1) extending ventrally of medial margin of lower face

(2) absent

√*4: Preorbital carina length

(0) extending dorsally to the ventral margin of the anterior ocellus

(1) extending dorsally to ventral margin of the antennal foramen

(2) Absent

√5: Upper face sculpture

(0) punctate and foveate

(1) foveate

(2) smooth

√6: Malar space length vs. half compound eye height (male)

(0) as long as or longer than half compound eye height

(1) shorter than half compound eye height

√*7: Ocellar ocular line length vs. lateral ocellus diameter

(0) shorter than lateral ocellus diameter

(1) as long or longer than lateral ocellus diameter

8: Posterior ocellar length vs. lateral ocellus diameter

(0) 1.5× as long as the diameter of the lateral ocellus

(1) 3× as long as the diameter of the lateral ocellus

*9: Ventral region of occipital carina curvature in lateral view

(0) curved

(1) straight

√10: Ventral region of the postoccipital carina shape

(0) raised

(1) not raised

11: Radicle color

(0) red

(1) black

(2) yellow

(3) orange

12: Radicle sculpture

(0) punctate

13: Scape color

(0) yellow

(1) orange

(2) black

(3) red

√*14: Scape length vs compound eye height

(0) scape longer than half compound eye height

(1) scape shorter than half compound eye height.

√15: Female flagellomere 1–8 shape

(0) distinctly wider than long (length of flagellomere < width of flagellomere)

(1) distinctly longer than wide (length of flagellomere > width of flagellomere)

√16: Shape of occiput

(0) as high as wide

(1) higher than wide

√17: Vertex sculpture

(0) foveate

(1) smooth

√18: Mandibular teeth number

(0) 2

(1) 4

√*19: Median notch of transverse pronotal carina presence

(0) absent

(1) present

(2) Transverse pronotal carina absent

√*20: Transverse pronotal carina length

(0) long, extending postero-laterally of epomia

(1) short, not extending postero-laterally of epomia

(2) absent

√21: Pronotal collar sculpture

(0) foveate

(1) scrobiculate

(2) scrobiculate and foveate

(3) smooth

√*22: Pronotal lobe carina presence

(0) present

(1) absent

√*23: Mesoscutum shape

(0) wider than long (length of mesoscutum < width of mesoscutum)

(1) as long as wide (length of mesoscutum > width of mesoscutum)

√*24: Antero-admedian line length vs. lateral ocellus diameter

(0) greater than lateral ocellus diameter

(1) equal to lateral ocellus diameter

(2) Decreased length

√*25: Parapsidal signum conspicuousness

(0) conspicuous

(1) inconspicuous

(2) absent

√26: Parapsidal signum presence

(0) present

(1) absent

√*27: Foveae on notaulus presence

(0) absent

(1) present

(2) smooth

√*28: Distance between depressions vs. diameter of depressions on internotaular area

(0) greater than the diameter of one depression

(1) less than the diameter of one depression

(2) Sparse

*29: Mesofemoral depression sculpture

(0) foveate

(1) smooth

*30: Mesofemoral depression pilosity presence

(0) present

(1) absent

*31: Ventral area of the mesopectus sculpture

(0) foveate

(1) smooth

(2) areolate

√*32: Medial region of transmetapectal carina presence

(0) present

(1) absent

√*33: Area dorsal of transmetapectal carina sculpture

(0) smooth

(1) areolate

√*34: Posterior propodeal projection shape in lateral view

(0) raised

(1) not raised

√*35: Posterior region of plica presence

(0) present

(1) absent

√*36: Dorsal area of the metapectal-propodeal complex sculpture

(0) areolate

(1) foveate

(2) smooth

√*37: Posterior margin of the metapectal-propodeal complex ventrally of the propodeal foramen curvature in lateral view

(0) straight

(1) curved

38: Mesosoma color

(0) red

(1) black

(2) black posteroventrally, red anterodorsally

(3) black posteroventrally, yellow anterodorsally

39: Metasoma color

(0) black

√*40: Dorsal region of petiole sculpture

(0) foveate

(1) wrinkled

(2) smooth

41: Fore leg color

(0) yellow

(1) red

42: Mid leg color

(0) yellow

(1) red

43: Hind leg color

(0) black

(1) red

√44: Metafemur length vs. metatibia length

(0) metafemur longer than metatibia

(1) metafemur equal to or shorter than metatibia

√45: Spines on posterior area of metatibia presence

(0) absent

(1) present

46: Metatibia length vs metabasitarsus length

(0) equal

(1) metatibia 1.4× to 1.6× as long as metabasitarsus

(2) metatibia 1.2× to 1.4× as long as metabasitarsus

(3) metatibia 2× as long as metabasitarsus

*47: Fore wing vein color

(0) black

(1) yellow

*48: Setae on proximal region of fore wing color

(0) black

(1) yellow

√*49: Mesosternum length vs. metasternum length

(0) ventral margin of mesosternum length equal to ventral margin of metapectus length

(1) ventral margin of mesosternum length longer than ventral margin of metapectus length

√50: Fore wing RS+M vein presence

(0) present

(1) absent

√51: Fore wing cell count

(0) 7 cells

(1) 6 cells

(2) 3 cells

(3) 1 cell

√52: Carinae on gena presence

(0) present

(1) absent

53: Mesoscutellum sculpture

(0) foveate

(1) smooth

54: Metanotum sculpture

(0) scrobiculate

*55: Ventral area of the metapectus sculpture

(0) entire area foveate

(1) dorsal half foveate and antero-ventral region smooth

56: Fore wing length

(0) extending beyond posterior margin of metasoma

## Appendix B

**Table:** Character descriptions in Manchester syntax.

“Notaulus texture”	“notaulus absent”	*has_part* only (not (notaulus))
“Notaulus texture”	“foveate”	*has_part* some (notaulus and (*is bearer of* some foveate))
“Notaulus texture”	“smooth”	*has_part* some (notaulus and (*is bearer of* some smooth))
“Median notch of transverse pronotal carina presence”	“absent”	*has_part* some (transverse pronotal carina and (*has_part* some (medial region and (*has_part* only (not (notch))))))
“Median notch of transverse pronotal carina presence”	“Transverse pronotal carina absent”	*has_part* only (not (transverse pronotal carina))
“Median notch of transverse pronotal carina presence”	“present”	*has_part* some (transverse pronotal carina and (*has_part* some (medial region and (*has_part* some notch))))
“Dorsal region of petiole sculpture”	“foveate”	*has_part* some (abdominal segment 2 and (*has_part* some (dorsal side and (*is bearer of* some foveate))))
“Dorsal region of petiole sculpture”	“smooth”	*has_part* some (abdominal segment 2 and (*has_part* some (dorsal side and (*is bearer of* some smooth))))
“Dorsal region of petiole sculpture”	“wrinkled”	*has_part* some (abdominal segment 2 and (*has_part* some (dorsal side and (*is bearer of* some wrinkled))))
“Setae on proximal region of fore wing color”	“yellow”	*has_part* some (fore wing and (*has_part* some (proximal region and (*has_part* some (seta and (*is bearer of* some yellow))))))
“Setae on proximal region of fore wing color”	“black “	*has_part* some (fore wing and (*has_part* some (proximal region and (*has_part* some (seta and (*is bearer of* some black))))))
“Pronotal collar sculpture”	“smooth”	*has_part* some (pronotal collar and (*is bearer of* some smooth))
“Pronotal collar sculpture”	“scrobiculate “	*has_part* some (pronotal collar and (*is bearer of* some scrobiculate))
“Pronotal collar sculpture”	“foveate”	*has_part* some (pronotal collar and (*is bearer of* some foveate))
“Pronotal collar sculpture”	“scrobiculate and foveate”	*has_part* some (pronotal collar and (*is bearer of* some scrobiculate) and (*is bearer of* some foveate))
“Preorbital carina length”	“Absent”	*has_part* only (not (preorbital carina))
“Preorbital carina length”	“extending dorsally to the ventral margin of the anterior ocellus”	*has_part* some (preorbital carina and (*has_part* some (dorsal margin and (*ventral_to* some (ventral margin and (*part_of* some compound eye))))))
“Preorbital carina length”	“extending dorsally to ventral margin of the antennal foramen”	*has_part* some (preorbital carina and (*has_part* some (dorsal margin and (*ventral_to* some (ventral margin and (*part_of* some torulus))))))
“Posterior propodeal projection shape in lateral view”	“not raised”	*has_part* some (posterior propodeal projection and (*is bearer of* some flat))
“Posterior propodeal projection shape in lateral view”	“raised “	*has_part* some (posterior propodeal projection and (*is bearer of* some raised))
“Upper face sculpture”	“smooth”	*has_part* some (upper face and (*is bearer of* some smooth))
“Upper face sculpture”	“punctate and foveate”	*has_part* some (upper face and (*is bearer of* some punctate) and (*is bearer of* some foveate))
“Upper face sculpture”	“foveate”	*has_part* some (upper face and (*is bearer of* some foveate))
“Parapsidal signum conspicuousness”	“conspicuous”	*has_part* some (parapsidal line and (*is bearer of* some conspicuous))
“Parapsidal signum conspicuousness”	“inconspicuous”	*has_part* some (parapsidal line and (*is bearer of* some inconspicuous))
“Parapsidal signum conspicuousness”	“absent”	*has_part* only (not (parapsidal line))
“Mesoscutum shape”	“as long as wide (length of mesoscutum > width of mesoscutum)”	*has_part* some (mesoscutum and (*is bearer of* some (square or elongated)))
“Mesoscutum shape”	“wider than long (length of mesoscutum < width of mesoscutum)”	*has_part* some (mesoscutum and (*is bearer of* some broad))
“Scape length vs compound eye height”	“scape shorter than half compound eye height”	*has_part* some (scape and (*is bearer of* some (length and (*has_measurement* some (((*has_unit* some length) and (*inheres in* some compound eye)) and (*has_magnitude* some float[< 0.5f]))))))
“Scape length vs compound eye height”	“scape longer than half compound eye height”	*has_part* some (scape and (*is bearer of* some (length and (*has_measurement* some (((*has_unit* some length) and (*inheres in* some compound eye)) and (*has_magnitude* some float[> 0.5f]))))))
“Malar space length vs. half compound eye height (male)”	“as long as or longer than half compound eye height”	*has_part* some (malar area and (*is bearer of* some (length and (*has_measurement* some ((*has_unit* some (length and (*inheres in* some compound eye))) and (*has_magnitude* some float[>= 0.5f]))))))
“Malar space length vs. half compound eye height (male)”	“shorter than half compound eye height”	*has_part* some (malar area and (*is bearer of* some (length and (*has_measurement* some ((*has_unit* some (length and (*inheres in* some compound eye))) and (*has_magnitude* some float[< 0.5f]))))))
“Subantennal carina length”	“extending dorsally of medial margin of lower face”	*has_part* some (subantennal carina and (*has_part* some (ventral margin and (*dorsal_to* some (ventral margin and (*part_of* some compound eye))))))
“Subantennal carina length”	“absent”	*has_part* only (not (subantennal carina))
“Subantennal carina length”	“extending ventrally of medial margin of lower face”	*has_part* some (subantennal carina and (*has_part* some (ventral margin and (*ventral_to* some (ventral margin and (*part_of* some compound eye))))))
“Ocellar ocular line length vs. lateral ocellus diameter”	“shorter than lateral ocellus diameter”	*has_part* some (ocular ocellar line and (*is bearer of* some (length and (*decreased_in_magnitude_relative_to* some (length and (*inheres in* somelateral ocellus))))))
“Ocellar ocular line length vs. lateral ocellus diameter”	“as long or longer than lateral ocellus diameter”	*has_part* some (ocular ocellar line and (*is bearer of* some (length and ((*increased_in_magnitude_relative_to* some (length and (*inheres in* somelateral ocellus))) or (*similar_in_magnitude_relative_to* some (length and (*inheres in* some lateral ocellus)))))))
“Female flagellomere 1–8 shape “	“distinctly longer than wide (length of flagellomere > width of flagellomere)”	(*has_part* some (first flagellomere and (*is bearer of* some elongated))) and (*has_part* some (second flagellomere and (*is bearer of* someelongated))) and (*has_part* some (fifth flagellomere and (*is bearer of* some elongated))) and (*has_part* some (third flagellomere and (*is bearer of*some elongated))) and (*has_part* some (fourth flagellomere and (*is bearer of* some elongated))) and (*has_part* some (sixth flagellomere and (*is bearer of* some elongated))) and (*has_part* some (seventh flagellomere and (*is bearer of* some elongated))) and (*has_part* some (eighth flagellomere and (*is bearer of* some elongated)))
“Female flagellomere 1–8 shape “	“distinctly wider than long (length of flagellomere < width of flagellomere)”	(*has_part* some (first flagellomere and (*is bearer of* some broad))) and (*has_part* some (second flagellomere and (*is bearer of* some broad))) and (*has_part* some (fifth flagellomere and (*is bearer of* some broad))) and (*has_part* some (third flagellomere and (*is bearer of* some broad))) and (*has_part* some (fourth flagellomere and (*is bearer of* some broad))) and (*has_part* some (sixth flagellomere and (*is bearer of* some broad))) and (*has_part* some (seventh flagellomere and (*is bearer of* some broad))) and (*has_part* some (eighth flagellomere and (*is bearer of* some broad)))
“Antero-admedian line length vs. lateral ocellus diameter”	“absent”	*has_part* only (not (anteroadmedian line))
“Antero-admedian line length vs. lateral ocellus diameter”	“greater than lateral ocellus diameter”	*has_part* some (anteroadmedian line and (*is bearer of* some (length and (*increased_in_magnitude_relative_to* some (length and (*inheres in* somelateral ocellus))))))
“Antero-admedian line length vs. lateral ocellus diameter”	“equal to lateral ocellus diameter”	*has_part* some (anteroadmedian line and (*is bearer of* some (length and (*similar_in_magnitude_relative_to* some (length and (*inheres in* somelateral ocellus))))))
“Posterior margin of the metapectal-propodeal complex ventrally of the propodeal foramen curvature in lateral view”	“curved”	*has_part* some (metapectal-propodeal complex and (*has_part* some (posterior margin and (*is bearer of* some curved))))
“Posterior margin of the metapectal-propodeal complex ventrally of the propodeal foramen curvature in lateral view”	“straight”	*has_part* some (metapectal-propodeal complex and (*has_part* some (posterior margin and (*is bearer of* some straight))))
“Medial region of transmetapectal carina presence “	“present”	*has_part* some (transmetapectal carina and (*is bearer of* some undivided))
“Medial region of transmetapectal carina presence “	“absent”	*has_part* some (transmetapectal carina and (*is bearer of* some split))
“Area dorsal of transmetapectal carina sculpture”	“areolate”	*has_part* some (area and ((*is bearer of* some areolate) and (*dorsal_to* some transmetapectal carina)))
“Area dorsal of transmetapectal carina sculpture”	“smooth”	*has_part* some (area and ((*is bearer of* some smooth) and (*dorsal_to* some transmetapectal carina)))
“Metafemur length vs. metatibia length”	“metafemur longer than metatibia”	*has_part* some (metafemur and (*is bearer of* some (length and (*increased_in_magnitude_relative_to* some (length and (*inheres in* somemetatibia))))))
“Metafemur length vs. metatibia length”	“metafemur equal to or shorter than metatibia”	*has_part* some (metafemur and (*is bearer of* some (length and ((*decreased_in_magnitude_relative_to* some (length and (*inheres in* somemetatibia))) or (*similar_in_magnitude_relative_to* some (length and (*inheres in* some metatibia)))))))
“Ventral region of occipital carina curvature in lateral view”	“straight”	*has_part* some (occipital carina and (*has_part* some (ventral region and (*is bearer of* some straight))))
“Ventral region of occipital carina curvature in lateral view”	“curved”	*has_part* some (occipital carina and (*has_part* some (ventral region and (*is bearer of* some curved))))
“Pronotal lobe carina presence”	“absent”	*has_part* some (pronotal lobe and (*has_part* only (not (carina))))
“Pronotal lobe carina presence”	“present”	*has_part* some (pronotal lobe and (*has_part* some carina))
“Parapsidal signum presence”	“absent”	*has_part* only (not (parapsidal line))
“Parapsidal signum presence”	“present”	*has_part* some parapsidal line
“Transverse pronotal carina length”	“long, extending postero-laterally of epomia”	*has_part* some (transverse pronotal carina and (*has_part* some (posterior margin and (*posterior_to* some epomial carina))))
“Transverse pronotal carina length”	“absent”	*has_part* only (not (transverse pronotal carina))
“Transverse pronotal carina length”	“short, not extending postero-laterally of epomia”	*has_part* some (transverse pronotal carina and (*has_part* some (posterior margin and (not (*posterior_to* some epomial carina)))))
“Spines on posterior area of metatibia presence”	“absent”	*has_part* some (metatibia and (*has_part* some (posterior region and (*has_part* only (not (spur))))))
“Spines on posterior area of metatibia presence”	“present”	*has_part* some (metatibia and (*has_part* some (posterior region and (*has_part* some spur))))
“Metatibia length vs metabasitarsus length”	“metatibia 1.4x to 1.6x as long as metabasitarsus”	*has_part* some (metatibia and (*is bearer of* some (length and (*has_measurement* some (*has_magnitude* some float[>= 1.4f , <= 1.6f])) and (*has_unit* some (length and (*inheres in* some metabasitarsus))))))
“Metatibia length vs metabasitarsus length”	“equal “	*has_part* some (metatibia and (*is bearer of* some (length and (*similar_in_magnitude_relative_to* some (length and (*inheres in* somemetabasitarsus))))))
“Metatibia length vs metabasitarsus length”	“metatibia 1.2x to 1.4x as long as metabasitarsus”	*has_part* some (metatibia and (*is bearer of* some (length and (*has_measurement* some (*has_magnitude* some float[>= 1.2f , <= 1.4f])) and (*has_unit* some (length and (*inheres in* some metabasitarsus))))))
“Metatibia length vs metabasitarsus length”	“metatibia 2x as long as metabasitarsus”	*has_part* some (metatibia and (*is bearer of* some (length and (*has_measurement* some ((*has_unit* some (length and (*inheres in* somemetabasitarsus))) and (*has_magnitude* value 2.0f))))))
“Head color”	“ventral half of upper face and lower face color red”	(*has_part* some (lower face and (*is bearer of* some red))) and (*has_part* some (upper face and (*has_part* some (ventral region and (*is bearer of*some red)))))
“Head color”	“dorsal half of upper face and vertex color black”	(*has_part* some (upper face and (*has_part* some (dorsal region and (*is bearer of* some black))))) and (*has_part* some (vertex and (*is bearer of*some black)))
“Head color”	“black”	*has_part* some (head and (*is bearer of* some black))
“Head color”	“yellow”	*has_part* some (head and (*is bearer of* some yellow))
“Head color”	“ventral half of upper face and lower face color red or yellow”	(*has_part* some (lower face and (*is bearer of* some (red or yellow)))) and (*has_part* some (upper face and (*has_part* some (ventral region and (*is bearer of* some (red or yellow))))))
“Head color”	“orange”	*has_part* some (head and (*is bearer of* some orange))
“Mandibular teeth number”	“4”	*has_part* some (mandible and (*has component* exactly 4 tooth))
“Mandibular teeth number”	“2”	*has_part* some (mandible and (*has component* exactly 2 tooth))
“Vertex sculpture”	“foveate”	*has_part* some (vertex and (*is bearer of* some foveate))
“Vertex sculpture”	“smooth”	*has_part* some (vertex and (*is bearer of* some smooth))
“Carinae on gena presence”	“present”	*has_part* some (gena and (*has_part* some carina))
“Carinae on gena presence”	“absent”	*has_part* some (gena and (*has_part* only (not (carina))))
“Metasoma color”	“black”	*has_part* some (metasoma and (*is bearer of* some black))
“Fore leg color”	“red”	*has_part* some (fore leg and (*is bearer of* some red))
“Fore leg color”	“yellow “	*has_part* some (fore leg and (*is bearer of* some yellow))
“Mid leg color”	“red”	*has_part* some (mid leg and (*is bearer of* some red))
“Mid leg color”	“yellow”	*has_part* some (mid leg and (*is bearer of* some yellow))
“Hind leg color”	“red”	*has_part* some (hind leg and (*is bearer of* some red))
“Hind leg color”	“black”	*has_part* some (hind leg and (*is bearer of* some black))
“Mesosternum length vs. metasternum length”	“ventral margin of mesosternum length longer than ventral margin of metapectus length”	*has_part* some (mesopectus and (*has_part* some (ventral margin and (*is bearer of* some (length and (*increased_in_magnitude_relative_to* some (length and (*inheres in* some (ventral margin and (*part_of* some metapectus))))))))))
“Mesosternum length vs. metasternum length”	“ventral margin of mesosternum length equal to ventral margin of metapectus length “	*has_part* some (mesopectus and (*has_part* some (ventral margin and (*is bearer of* some (length and (*similar_in_magnitude_relative_to* some (length and (*inheres in* some (ventral margin and (*part_of* some metapectus))))))))))
“Fore wing RS+M vein presence”	“absent”	not (phenotype_9283)
“Fore wing RS+M vein presence”	“present”	*has_part* some (fore wing and (*has_part* some wing vein))
“Fore wing cell count”	“6 cells”	*has_part* some (fore wing and (*has component* exactly 6 cell))
“Fore wing cell count”	“7 cells”	*has_part* some (fore wing and (*has component* exactly 7 cell))
“Fore wing cell count”	“3 cells”	*has_part* some (fore wing and (*has component* exactly 3 cell))
“Fore wing cell count”	“1 cell”	*has_part* some (fore wing and (*has component* exactly 1 cell))
“Mandible color vs clypeus color”	“mandible color same as clypeus color”	*has_part* some (mandible and (*is bearer of* some (color and (*similar_in_magnitude_relative_to* some (color and (*inheres in* some clypeus))))))
“Mandible color vs clypeus color”	“mandible color different than clypeus color”	*has_part* some (mandible and (*is bearer of* some (color and (*different_in_magnitude_relative_to* some (color and (*inheres in* some clypeus))))))
“Mandible color vs clypeus color”	“black”	*has_part* some (mandible and (*is bearer of* some black))
“Scape color”	“orange”	*has_part* some (scape and (*is bearer of* some orange))
“Scape color”	“red”	*has_part* some (scape and (*is bearer of* some red))
“Scape color”	“black”	*has_part* some (scape and (*is bearer of* some black))
“Scape color”	“yellow”	*has_part* some (scape and (*is bearer of* some yellow))
“Radicle color”	“orange”	*has_part* some (radicle and (*is bearer of* some orange))
“Radicle color”	“black”	*has_part* some (radicle and (*is bearer of* some black))
“Radicle color”	“red”	*has_part* some (radicle and (*is bearer of* some red))
“Radicle color”	“yellow”	*has_part* some (radicle and (*is bearer of* some yellow))
“Radicle sculpture”	“punctate”	*has_part* some (radicle and (*is bearer of* some punctate))
“Posterior ocellar length vs. lateral ocellus diameter”	“1.5x as long as the diameter of the lateral ocellus “	*has_part* some (posterior ocellar line and (*is bearer of* some (length and (*has_measurement* some ((*has_unit* some (length and (*inheres in* somelateral ocellus))) and (*has_magnitude* value 1.5f))))))
“Posterior ocellar length vs. lateral ocellus diameter”	“3x as long as the diameter of the lateral ocellus”	*has_part* some (posterior ocellar line and (*is bearer of* some (length and (*has_measurement* some ((*has_unit* some (length and (*inheres in* somelateral ocellus))) and (*has_magnitude* value 3.0f))))))
“Ventral area of the mesopectus sculpture”	“foveate”	*has_part* some (mesopectus and (*has_part* some (ventral region and (*is bearer of* some foveate))))
“Ventral area of the mesopectus sculpture”	“smooth”	*has_part* some (mesopectus and (*has_part* some (ventral region and (*is bearer of* some smooth))))
“Ventral area of the mesopectus sculpture”	“areolate”	*has_part* some (mesopectus and (*has_part* some (ventral region and (*is bearer of* some areolate))))
“Mesofemoral depression sculpture”	“smooth”	*has_part* some (femoral depression and (*is bearer of* some smooth))
“Mesofemoral depression sculpture”	“foveate”	*has_part* some (femoral depression and (*is bearer of* some foveate))
“Mesofemoral depression pilosity presence”	“present”	*has_part* some (femoral depression and (*is bearer of* some setose))
“Mesofemoral depression pilosity presence”	“absent”	*has_part* some (femoral depression and (*is bearer of* some (pilosity and (not (setose)))))
“Ventral area of the metapectus sculpture”	“entire area foveate”	(*has_part* some metapectus) and (*has_part* some (ventral region and (*is bearer of* some foveate)))
“Ventral area of the metapectus sculpture”	“dorsal half foveate and antero-ventral region smooth”	(*has_part* some metapectus) and (*has_part* some (ventral region and (*has_part* some (antero-ventral region and (*is bearer of* some smooth))) and (*has_part* some (dorsal region and (*is bearer of* some foveate)))))
“Mesoscutellum sculpture”	“foveate”	*has_part* some (mesoscutellum and (*is bearer of* some foveate))
“Mesoscutellum sculpture”	“smooth”	*has_part* some (mesoscutellum and (*is bearer of* some smooth))
“Metanotum sculpture”	“scrobiculate”	*has_part* some (metanotum and (*is bearer of* some scrobiculate))
“Fore wing vein color”	“yellow”	*has_part* some (fore wing and (*has_part* some (wing vein and (*is bearer of* some yellow))))
“Fore wing vein color”	“black”	*has_part* some (fore wing and (*has_part* some (wing vein and (*is bearer of* some black))))
“Fore wing length”	“extending beyond posterior margin of metasoma”	*has_part* some (fore wing and (*is bearer of* some (length and (*increased_in_magnitude_relative_to* some (length and (*inheres in* somemetasoma))))))
“Posterior region of plica presence”	“present”	*has_part* some (plica and (*is bearer of* some (shape and (not (truncated)))))
“Posterior region of plica presence”	“absent”	*has_part* some (plica and (*is bearer of* some truncated))
“Distance between depressions vs. diameter of depressions on mesoscutum”	“less than the diameter of one depression”	*has_part* some (interstice and (*is bearer of* some (length and (*decreased_in_magnitude_relative_to* some (length and (*inheres in* some (depression and (*part_of* some mesoscutum))))))))
“Distance between depressions vs. diameter of depressions on mesoscutum”	“smooth”	*has_part* some (mesoscutum and (*is bearer of* some smooth))
“Distance between depressions vs. diameter of depressions on mesoscutum”	“greater than the diameter of one depression”	*has_part* some (interstice and (*is bearer of* some (length and (*increased_in_magnitude_relative_to* some (length and (*inheres in* some (depressionand (*part_of* some mesoscutum))))))))
“Dorsal area of the metapectal-propodeal complex sculpture”	“foveate”	*has_part* some (metapectal-propodeal complex and (*has_part* some (dorsal side and (*is bearer of* some foveate))))
“Dorsal area of the metapectal-propodeal complex sculpture”	“areolate”	*has_part* some (metapectal-propodeal complex and (*has_part* some (dorsal side and (*is bearer of* some areolate))))
“Dorsal area of the metapectal-propodeal complex sculpture”	“smooth”	*has_part* some (metapectal-propodeal complex and (*has_part* some (dorsal side and (*is bearer of* some smooth))))
“Ventral region of the postoccipital carina shape”	“raised”	*has_part* some (postoccipital carina and (*has_part* some (ventral region and (*is bearer of* some raised))))
“Ventral region of the postoccipital carina shape”	“not raised”	*has_part* some (postoccipital carina and (*has_part* some (ventral region and (*is bearer of* some flat))))
“Shape of occiput”	“as high as wide”	*has_part* some (occiput and (*is bearer of* some circular))
“Shape of occiput”	“higher than wide”	*has_part* some (occiput and (*is bearer of* some oblong))
“Mesosoma color”	“red”	*has_part* some (metafemur and (*is bearer of* some red))
“Mesosoma color”	“black posteroventrally, red anterodorsally”	*has_part* some (metafemur and (*has_part* some (antero-dorsal region and (*is bearer of* some red))) and (*has_part* some (postero-ventral regionand (*is bearer of* some black))))
“Mesosoma color”	“black posteroventrally, yellow anterodorsally”	*has_part* some (metafemur and (*has_part* some (antero-dorsal region and (*is bearer of* some yellow))) and (*has_part* some (postero-ventral regionand (*is bearer of* some black))))
“Mesosoma color”	“black”	*has_part* some (metafemur and (*is bearer of* some black))

## Appendix C

**Table:**

*Evaniscus lansdownei * Mullins, 2012	NCSU 33809, NCSU 0067242
*Evaniscus rafaeli * Kawada, 2012	NCSU 0067243–0067246
*Evaniscus rufithorax * Enderlein, 1905	NCSU 0002391–0002400, NCSU 0006967–0007000, NCSU 0009886–0009891, NCSU 18832, NCSU 0018399, NCSU 0041750, NCSU 0018398, NCSU 41741, NCSU 41742, NCSU 33582, NCSU 0067257–0067290, NCSU 0036699, mx_id: 15337, 15338, 15340–15342, 15349, 15350, 15351, 23037
*Evaniscus marginatus * (Cameron, 1887)	NCSU 0009892–0009894, NCSU 53033, NCSU 0067240, 0067241, NCSU 0041748, mx_id: 15343–15346, 15348
*Evaniscus tibialis * Szépligeti, 1903	NCSU 0009896, NCSU 0041745–0041747, NCSU 0067247–0067256, mx_id: 15339, 15347, 15132
*Evaniscus sulcigenis * Roman, 1917	NCSU 0009895
