# Corrigenda: Mullins PL, Kawada R, Balhoff JP, Deans AR (2012) A revision of *Evaniscus* (Hymenoptera, Evaniidae) using ontology-based semantic phenotype annotation. ZooKeys 223: 1–38, doi: 10.3897/zookeys.223.3572

**DOI:** 10.3897/zookeys.278.5108

**Published:** 2013-03-21

**Authors:** Patricia L. Mullins, Rico Kawada, James P. Balhoff, Andrew R. Deans

**Affiliations:** 1 Department of Entomology, North Carolina State University, Campus Box 7613, 2301 Gardner Hall, Raleigh, NC 27695-7613 USA; 2 Department of Ecology, Evolution, and Organismal Biology, 251 Bessey Hall, Iowa State University, Ames, IA 50011 USA; 3 Museu de Zoologia da Universidade de São Paulo. Av. Nazaré, 481, Ipiranga, CEP 04263-000, São Paulo-SP, Brazil; 4 National Evolutionary Synthesis Center, Durham, NC USA; 5 Department of Biology, University of North Carolina at Chapel Hill, Chapel Hill, NC USA; 6 Department of Entomology, Pennsylvania State University, 501 ASI Building, University Park, PA 16802 USA

Several errors came to our attention after our manuscript was published, which we address here. First, we uploaded the wrong table for Appendix C, which was supposed to have verbose descriptions of material examined. We also neglected to provide complete repository names for the museum codens. We regret these errors and provide these data here in Table 1 and in an updated Appendix C.

At least one reader was confused about our observations of type specimens and the subsequent designation of *Evaniscus sulcigenis* Roman, 1917 as a junior subjective synonym of *Evaniscus rufithorax* Enderlein, 1905. We observed the type specimen of *Evaniscus sulcigenis* directly (specimen data in the corrected Appendix C below; see also Figs 1, 2) and neglected to include that declaration and the listing of *Evaniscus sulcigenis* as a junior subjective synonym in the taxonomic treatment of *Evaniscus rufithorax*.

**Table 1.** Specimen repositories from Mullins et al. 2012.

**Coden**	**Repository name, address**	**Facilitator**
AEIC	American Entomological Institute, Gainesville, FL, USA	David Wahl
BMNH	The Natural History Museum [formerly British Museum (Natural History)], London, UK	David Notton
NCSU	NCSU Insect Museum, North Carolina State University, Raleigh, NC, USA	Bob Blinn
HNHM	Hungarian Natural History Museum, Budapest, Hungary	Csősz Sándor
INPA	Instituto Nacional de Pesquisas da Amazoonia, Colecão Sistemática da Entomologia, Manaus, Amazonas, Brazil	Augusto Loureiro Henriques
MPEG	Museu Paraense Emilio Goeldi, Belém, Pará, Brazil	Orlando Tobias Silveira
ZMPA	Polish Academy of Science, Museum of the Institute of Zoology, Warsaw, Poland	Wioletta Tomaszewska
USNM	National Museum of Natural History [formerly United States National Museum], Washington, DC, USA	David Smith
NHRS	Naturhistoriska Riksmuseet, Stockholm, Sweden	Bert Viklund
ZMHB	Museum für Naturkunde der Humboldt-Universität, Berlin, Germany	Frank Koch
MZSP	Museu de Zoologia da Universidade de São Paulo, São Paulo, Brazil	Carlos Roberto Ferreira Brandão
MIUP	Universidad de Panamá, Museo de Invertebrados Graham B. Fairchild, Panama City, Panama	Diomedes Quintero
CAS	California Academy of Sciences, San Fransisco, CA, USA	Robert Zuparko
MZLU	Museum of Zoology, Lund University, Sweden	Roy Danielsson
UCDC	Bohart Museum, University of California, Davis, CA, USA	Steve Heydon
INHS	Illinois Natural History Survey, Champaign, IL, USA	Colin Favret (now Dmitry A. Dmitriev)
TAMU	Texas A & M University Insect Collection, College Station, TX, USA	Edward Riley
INBC	Instituto Nacional de Biodiversidad, Santo Domingo de Heredia, Costa Rica	Carlos Víquez
IAVH	Instituto de Investigación de Recursos Biológicos Alexander von Humboldt, Bogotá D.C., Colombia	Fernando Fernandez

Finally, a number of typos persisted in the manuscript despite our intention to properly vet the taxonomic treatments. We offer our corrections to help resolve any confusion:

Our database had two entries for each type specimen, and so the type data was listed twice in each treatment. This error has been fixed in the database.

*Pseudevania* is not a misspelling of *Evaniscus* but rather a junior objective synonym (Deans 2005). This error has been fixed in the database.

The type specimens for *Evaniscus marginata* (Cameron, 1887) and *Evaniscus tibialis* Szépligeti 1903 are not necessarily holoypes. Neither Cameron nor Szépligeti explicitly designate holotypes, nor did they list all specimens examined in their treatments of these species. Each type specimen is deposited at the taxonomist’s home institution (BMNH for Cameron and HNHM for Szépligeti), and we are unaware of any other specimens that would have been available for observation at the time.

The repository for the holotype of *Evaniscus lansdownei* Mullins should be IAVH, not NCSU.

**Figure 1.**
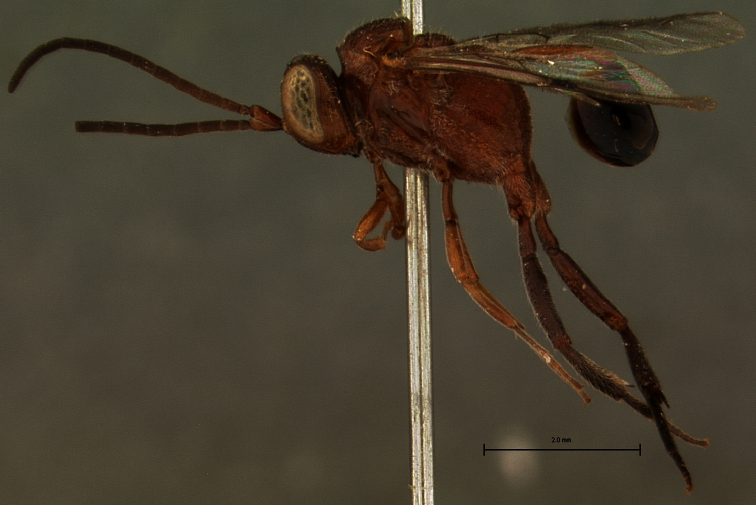
*Evaniscus sulcigenis* Roman, 1917. Lateral habitus (whole body) of holotype.

**Figure 2.**
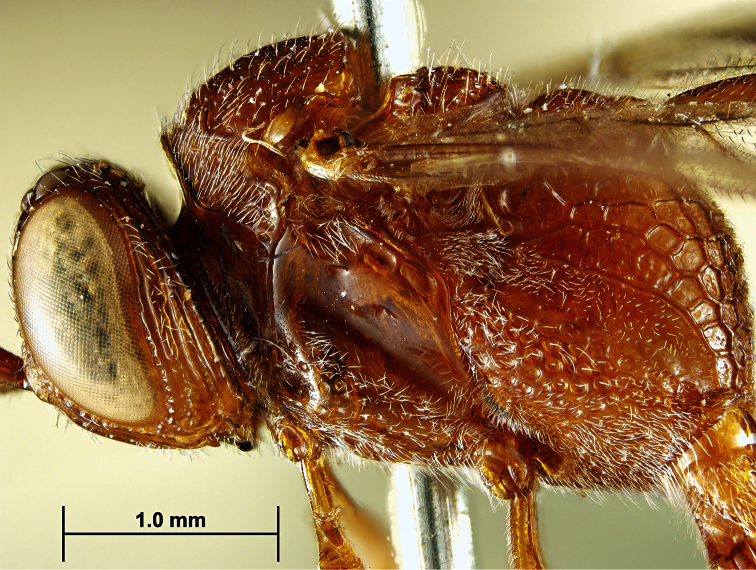
*Evaniscus sulcigenis* Roman, 1917. Lateral habitus (mesosma and head) of holotype.

## Figures and Tables

**Table:** *Evaniscus lansdownei* Mullins, 2012.

**Identifier(s)**	**Repository**	**Sex**	**Collecting event**
NCSU 33809	IAVH	male	COLOMBIA-AMAZONAS Mata Mata Sta., Tierra Firme Mar 8-12 2000 Sweep, Sharkey {HOLOTYPE}
NCSU 67242	INPA	male	BRASIL: Amazonas Balawa-u, 30Km NW of Parque Nacional Parima Tapirapecó -1.8069, -63.7844 14.ix.1995 Malaise LS Aquino col {PARATYPE}.

**Table:** *Evaniscus marginatus* (Cameron, 1887).

**Identifier(s)**	**Repository**	**Sex**	**Collecting event**
NCSU 53033; DERV 052	INBC	male	Costa Rica: Prov. Guanacaste: Bosque N. Diriá, Retallano, Alred. Torre de Control de Incendio: 600-700m, 15.xi-16.xii.2001, I. Jimenez, Malaise L_N_238550_358650 #66658
NCSU 9894	TAMU	female	Mexico: Oaxaca, 9 miles ne. Mitla July 20, 1985 Jones, Schaffner
NCSU 9893	UCDC	male	Costa Rica: Guanacaste Prov. near Cañas, 19.VIII.1992 F. D. Parker, MT
NCSU 9892	AEIC	female	S. Rosa Park, Guan., C. Rica 22 Feb. 77 D. H. Janzen, riparian
NCSU 41748	USNM	female	ECUADOR: NAPO Res. Ethnica Waorani, 1km S. Onkone Gare Camp, Trans.Ent. 12 Jan. 1994 220m 00°39’10’’S 076°26’’W T.L. Erwin, et. al Insecticidal fogging of mostly bare green leaves, some with covering of lichenous or bryophytic plants in terre firme forest. At Trans. 10, Sta. 2 Project MAXUS Lot 562 USNM 2027843
NCSU 67240	MZSP	female	BRASIL Piauí Floriano -6.7651, -43.0257 05-12.xi.1992 Malaise Amarante, Brandão, Cancello, Moutinho & Ponte, col.
	BMNH	female	COSTA RICA: Guanacaste Pv. Sta. Rosa NP Sn. Emilio-8-C Janzen & Gauld 10-31.i.1987
	BMNH	female	COSTA RICA: Guanacaste Pv. Sta. Rosa NP Sn. Emilio-8-C Janzen & Gauld 2-23.iii.1986
	BMNH	female	COSTA RICA: Guanacaste Pv. Sta. Rosa NP Sn. Emilio-6-C Janzen & Gauld 13.vii-3.viii.85
	BMNH	female	COSTA RICA: Guanacaste Pv. Sta. Rosa NP Bosq. Hum-12-C Janzen & Gauld 29.xi.-20.xii.1986
NCSU 67241	MZSP	female	BRASIL Piauí Floriano -6.7651, -43.0257 05-12.xi.1992 Malaise Amarante, Brandão, Cancello, Moutinho & Ponte, col.

**Table:** *Evaniscus rafaeli* Kawada, 2012.

**Identifier(s)**	**Repository**	**Sex**	**Collecting event**
NCSU 67243	INPA	female	BRASIL Amazonas Manaus Reserva [Florestal Adolpho] Ducke -2.9558, -59.9222 07-21.xi.1994 Arm. suspensa 20m, Torre JA Rafael & J Vidal col. {PARATYPE}
NCSU 67244	MZSP	female	BRASIL Amazonas Manaus Reserva [Florestal Adolpho] Ducke -2.9558, -59.9222 06-20.x.1994 Arm. suspensa 10m, Torre JA Rafael & J Vidal col. {PARATYPE}
NCSU 67245	INPA	female	BRASIL Amazonas Manaus Reserva [Florestal Adolpho] Ducke, 26 Km NE Manaus -2.9558, -59.9222 22.ix.1988 Arm. suspensa 21m JA Rafael col. {HOLOTYPE}
NCSU 67246	INPA	female	BRASIL Amazonas Manaus Reserva [Florestal Adolpho] Ducke, 26 Km NE Manaus -2.9558, -59.9222 06.x.1988 Arm. suspensa 15m JA Rafael col. {PARATYPE}

**Table:** *Evaniscus rufithorax* Enderlein, 1905.

**Identifier(s)**	**Repository**	**Sex**	**Collecting event**
NCSU 41750	AEIC	male	Amazonas, Brazil 71°38'W, 4°33'S IX.1979 Alvarenga
DERV 010b	AEIC		Amazonas, Brazil 71°38'W, 4°33'S IX.1979 Alvarenga
NCSU 2400	AEIC	male	Sinop, M. Grosso 12°31'S, 55°37'W, Oct.1976 Brazil M. Alvarenga
NCSU 2399	AEIC	male	Sinop, M. Grosso 12°31'S, 55°37'W, Oct.1976 Brazil M. Alvarenga
NCSU 2398	AEIC	male	Sinop, M. Grosso 12°31'S, 55°37'W, Oct.1976 Brazil M. Alvarenga
NCSU 2397	AEIC	male	Sinop, M. Grosso 12°31'S, 55°37'W, Oct.1976 Brazil M. Alvarenga
NCSU 2396	AEIC	male	Sinop, M. Grosso 12°31'S, 55°37'W, X.1974 Brazil M. Alvarenga
NCSU 2395	AEIC	male	Sinop, M. Grosso 12°31'S, 55°37'W, X.1974 Brazil M. Alvarenga
NCSU 2394	INHS	male	Peru: Madre de Dios: Tambopata Preserve, 270m, 22-30-V-1995 S. Cameron, J. Whitfield, rainforest
NCSU 2393	INHS	male	Peru: Madre de Dios: Tambopata Preserve, 270m, 22-30-V-1995 S. Cameron, J. Whitfield, rainforest
NCSU 2392	INHS	male	Peru: Madre de Dios: Tambopata Preserve, 270m, 22-30-V-1995 S. Cameron, J. Whitfield, rainforest
NCSU 2391	INHS	male	Peru: Madre de Dios: Tambopata Preserve, 270m, 22-30-V-1995 S. Cameron, J. Whitfield, rainforest
NCSU 7000	AEIC	female	Napo & Coca Rivers, V.2-10-65 Ecuador, Luis Peña
NCSU 6999	AEIC	male	Napo & Coca Rivers, V.2-10-65 Ecuador, Luis Peña
NCSU 6998	AEIC	male	Napo & Coca Rivers, V.2-10-65 Ecuador, Luis Peña
NCSU 6997	AEIC	male	Napo & Coca Rivers, V.2-10-65 Ecuador, Luis Peña
NCSU 6996	AEIC	female	Napo & Coca Rivers, V.2-10-65 Ecuador, Luis Peña
NCSU 6995	AEIC	male	Napo & Coca Rivers, V.2-10-65 Ecuador, Luis Peña
NCSU 6994	AEIC	male	Napo & Coca Rivers, V.2-10-65 Ecuador, Luis Peña
NCSU 6993	AEIC	male	Coca & Napo Rivers, V.1-12.65 Ecuador Luis Peña
NCSU 6992	AEIC	female	Coca & Napo Rivers, V.1-12.65 Ecuador Luis Peña
NCSU 6991	AEIC	male	Cumbaratza (E) XI.21.70 Ecuador Luis E. Peña
NCSU 6990	AEIC	female	Coca Ecuador May 1965 Luis Peña
NCSU 6989	AEIC	male	Coca Ecuador May 1965 Luis Peña
NCSU 6988	AEIC	male	Coca Ecuador May 1965 Luis Peña
NCSU 6987	AEIC	male	Coca Ecuador May 1965 Luis Peña
NCSU 6986	AEIC	male	Coca Ecuador May 1965 Luis Peña
NCSU 6985	UCDC	male	Ecuador: Prov. Mor. Santiago, Miazal 50km se. Macas, 4-7.I.1993 300m M. & J. Wasbauer
NCSU 6984	UCDC	female	Ecuador: Prov. Mor. Santiago, Miazal 50km se. Macas, 4-7.I.1993 300m M. & J. Wasbauer
NCSU 6983	UCDC	male	Ecuador: Prov. Mor. Santiago, Miazal 50km se. Macas, 4-7.I.1993 300m M. & J. Wasbauer
NCSU 6982	UCDC	male	Ecuador: Prov. Mor. Santiago, Miazal 50km se. Macas, 4-7.I.1993 300m M. & J. Wasbauer
NCSU 6981	UCDC	female	Ecuador: Prov. Mor. Santiago, Miazal 50km se. Macas, 4-7.I.1993 300m M. & J. Wasbauer
NCSU 6980	UCDC	female	Ecuador: Prov. Mor. Santiago, Miazal 50km se. Macas, 4-7.I.1993 300m M. & J. Wasbauer
NCSU 6979	UCDC	male	Ecuador: Prov. Mor. Santiago, Miazal 50km se. Macas, 4-7.I.1993 300m M. & J. Wasbauer
NCSU 6978	USNM	male	Ecuador: Napo, Res. Ethnica Waorani, 1km S. Onkone Gare Camp, Trans. Ent. 30Jun 1995, 220m, 00°39' 10”S, 076°26'W, T. L. Erwin et al. insecticidal fogging of mostly bare green leaves, some with covering of lichenous or bryophytic plants in terre firma forest, At trans.8, Sta. 9 Project MAXUS Lot 1079
NCSU 6977	AEIC	female	Ecuador: Napo forest, E. Coca, March 6, 1983, Lars Huggert
NCSU 6976	AEIC	male	Ecuador: Napo forest, E. Coca, March 6, 1983, Lars Huggert
NCSU 6975	INHS	male	Ecuador: Orellana, Yasuni Biological Station, 3-7 VII-2000, S. Cameron, J. Whitfield
NCSU 6974	AEIC	male	Ecuador: Napo, S. Sacha, III.7.83, Lars Huggert
NCSU 6973	AEIC	male	Ecuador: Napo, Sacha, III.5.83, Lars Huggert
NCSU 6972	AEIC	male	Ecuador: Napo, P. Misahuelli, Feb. 20, 1983, Lars Huggert
NCSU 6971	AEIC	male	Ecuador: Napo, Tena, II.16.83, Lars Huggert
NCSU 6970	AEIC	male	Ecuador: Napo, Tena, II.16.83, Lars Huggert
NCSU 6969	AEIC	female	Ecuador: Napo, Sacha, III.7.83, Lars Huggert
NCSU 6968	AEIC	male	Amazonas, Brazil 71°38'W, 4°33'S IX.1979 Alvarenga
NCSU 6967	AEIC	female	Tucuruí, Pará I.1979 Brazil M. Alvarenga
NCSU 9891	AEIC	male	Avispas, Perú 30m nr. Marcapata Oct. 20-30, 1962 Luis Peña
NCSU 9890	AEIC	female	Avispas, Perú 30m nr. Marcapata Sept. 1962 Luis Peña
NCSU 9889	UCDC	male	Brazil: RO Fazenda Rancho Grande 62km s. Ariquemes, 12-22.XI.1991 E. M. Fischer
NCSU 9888	UCDC	male	Brazil: RO Fazenda Rancho Grande 62km s. Ariquemes, 12-22.XI.1991 E. M. Fischer
NCSU 9887	AEIC	male	Peru: Loreto Iquitos II.7.84 Lars Huggert
NCSU 9886	AEIC	male	Peru: Junin Satipo I-23-84 Lars Huggart
NCSU 18399	MZLU	male	Peru: Junin Satipo I-23-84 Lars Huggart
NCSU 18398	MZLU	female	Peru: Junin, Satipo 21-I-1984 leg. Lars Huggert
NCSU 41741	NCSU	male	COLOMBIA Putumayo PNN La Paya Bocana Mamansoya 0°6'S, 74°58'W, 330m Red 23.ix.2001 D. Campos, leg. M.2092
NCSU 41742	NCSU	male	COLOMBIA Putumayo PNN La Paya Bocana Mamansoya 0°6'S, 74°58'W, 330m Red 23.ix.2001 D. Campos, leg. M.2092
NCSU 33582	NCSU	male	COLOMBIA Putumayo PNN La Paya Cabana La Paya 0°2'S, 75°12'W, 330m Red. 25.ix.2001 D. Campos, leg. M.2083
NCSU 67257	INPA	male	BRASIL Amazonas S. Izadel. R. Negro, Maturaca 11-13.x.1990 Malaise JA Rafael col.
NCSU 67258	INPA	male	BRASIL Roraima Ilha de Maracá Rio Ura 3.2589 -60.9889 21-30.xi.1987 fogging JA Rafael & equipe col.
NCSU 67259	INPA	male	BRASIL Pará Alter do Chão 09-11.ix.1991 Malaise JA Rafael col.
NCSU 67260	INPA	male	BRASIL Rondônia Nova Mamoré Parque Estadual de Guajará Mirim, Rio Formoso, Terra firme -10.3239, -64.6578 22.x.1995 J Vidal & LS Aquino col.
NCSU 67261	INPA	male	BRASIL Amazonas Tabatinga 13-17.iv.1992 Malaise J Vidal & Lilian col.
NCSU 67262	INPA	male	BRASIL Amazonas Tabatinga 13-17.iv.1992 Malaise J Vidal & Lilian col.
NCSU 67263	INPA	male	BRASIL Amazonas Tabatinga 13-17.iv.1992 Malaise J Vidal & Lilian col.
NCSU 67264	INPA	male	BRASIL Amazonas Tabatinga 13-17.iv.1992 Malaise J Vidal & Lilian col.
NCSU 67265	INPA	female	BRASIL Amazonas Manaus ZF03 Km 23, Fazenda Estelo, Reserva 1208 (RLE) -2.3761, -59.8775 viii.1986 Malaise Bert Klein col.
NCSU 67266	INPA	female	BRASIL Amazonas Borba Rio Abacaxis, Paxiúba -4.4800, -58.5733 02-04.vi.2008 Malaise JA Rafael & equipe col.
NCSU 67267	INPA	male	BRASIL Amazonas Manaus Reserva [Florestal Adolpho] Ducke, Igarapé Acará, Rod[ovia] AM-010, Km 26 x.2001 Malaise JF Vidal
NCSU 67268	INPA	male	BRASIL Amazonas Manaus Reserva [Florestal Adolpho] Ducke, Igarapé Tinga -2.9560, -59.9223 iii.2003 Malaise, JMF Ribeiro
NCSU 67269	INPA	female	BRASIL Amazonas Manaus Reserva [Florestal Adolpho] Ducke, Igarapé Tinga -2.9560, -59.9223 iii.2003 Malaise, JMF Ribeiro
NCSU 67270	INPA	female	BRASIL Amazonas Manaus Reserva [Florestal Adolpho] Ducke, Igarapé Ipiranga -2.9560, -59.9223 iv.2003 Malaise JMF Ribeiro
NCSU 67271	INPA	male	BRASIL Amazonas Parque Nacional do Jaú, Rio Unini -1.7317, -61.9089 20-24.vi.1996 Malaise AL Henriques, JF Vidal & FL Oliveira col.
NCSU 67272	INPA	male	BRASIL Amazonas Manaus Reserva [Florestal Adolpho] Ducke, Igarapé Acará -2.9560, -59.9223 05.viii.1993 Malaise JF Vidal col.
NCSU 67273	INPA	male	BRASIL Maranhão Serra Esplanada 07.xii.2001 Malaise JA Rafael & equipe col.
NCSU 67274	CAS	male	PERU Monzon Valley Tingo Maria 23.ix.1954 EI Schlinger & ES Ross col.
NCSU 67275	CAS	male	PERU Madre de Dios Rio Tambopata Reserve, 20 air Km SW of Puerto Maldonato, 290m 01-26.xi.1982 ER Ross col.
NCSU 67276	CAS	female	PERU Monzon Valley Tingo Maria 02.ix.1954 EI Schlinger & ES Ross col.
NCSU 67277	MIUP	male	PERU Madre de Dios Est. Pakitza, Res. Manu 10.vi-06.vii.1993 R Cambra & T Amarilla
NCSU 67278	MIUP	male	PERU Madre de Dios Est. Pakitza, Res. Manu 10.vi-06.vii.1993 R Cambra & T Amarilla
NCSU 67279	INPA	female	BRAZIL Amazonas [Manaus] AM: 010. Km. 31 Embrapa, colheita de cacau 27.iii.1991 Shannon (isca de fruta) LP Albuquerque & JE Binda col.
NCSU 67287	MZSP	male	BRASIL Rondônia Porto Velho, Rio Madeira 11-19.v.2010 RR Silva & RM Feitosa Malaise
NCSU 67288	MZSP	male	BRASIL Rondônia Porto Velho, Rio Madeira 11-19.v.2010 RR Silva & RM Feitosa Malaise
NCSU 67289	MZSP	male	BRASIL Rondônia Porto Velho, Rio Madeira 11-19.v.2010 RR Silva & RM Feitosa Malaise
NCSU 67290	MZSP	male	BRASIL Rondônia Porto Velho, Rio Madeira 11-19.v.2010 Light Trap F. Albertoni
NCSU 67280	MPEG	male	BRASIL Pará Benevides PA 408 Km 06 -1.3608, -48.2267 23.vi.1981 flight trap LS Gorayeb col.
NCSU 67281	MPEG	male	BRASIL Pará Xingu Rio Xingu, Ilha Boa Vista, 600 m da trilha -2.2000, -51.0167 20-24.xi.2007 Malaise
NCSU 67282	MPEG	male	BRASIL Pará Xingu Rio Xingu, Bom Jardim -2.3742, -51.011 22.xi.2007
NCSU 67283	MPEG	male	BRASIL Pará Primavera Quaitipura, Fazenda Feitosa -0.9419, -47.1178 28-30.xi.1992 armadilha suspensa (4 m) J Dias col.
NCSU 67284	MPEG	male	BRASIL Pará Juruti estrada p/o Mutum -2.1520, -56.0870 30.xi.2008 Malaise(4)
NCSU 67285	MPEG	female	BRASIL Pará Altamira Rio Xingu, A1. Itapuama, S. Antonio -7.5450, -54.7120 19-23.viii.2008 Malaise (4) OT Oliveira, SS Silva, JO Dias col.
NCSU 67286	MPEG	female	BRASIL Pará Xingu área- [Santo] Antonio -7.5450-54.7120 06-11.xi.2007 Malaise(1)
	BMNH	male	Suriname: Marowijne River vii.1965 E.A.M. Gale Cambridge Exped. B.M. 1965-516 E. rufithorax Enderlein 1905 det. by M.Huben 1995 ID#0301.17 (male) BMNH
	BMNH	male	MAZARUNI: 2nd growth (Low Forest) 16.viii.1937 BRITISH GUIANA: Coll. Richards & Smart B.M. 1937-776 Evaniscus rufithorax Enderlein 1905 det.M. Huben 1995 ID# 0301.18 (male) BMNH
	BMNH	male	PERU: Loreto Pucallpa 12.vi.1962 J.M. Schunke B.M. 1962-491 Evaniscus, Huben 1992
	BMNH	male	PERU: Loreto Pucallpa 5.viii.1963 J.M. Schunke B.M. 1962-565
	BMNH	male	Forested eastern foothills of the Andes, 2000 ft. PERU: Tingo Maria 1 km. E. of town Malaise trap, dense woodland 13.viii.1971
	ZMHB	male	Bolivia Mapiri Staudinger, V.
	ZMHB	male	Bolivia Mapiri Staudinger, V.
	ZMHB	male	Peru Pachitea-Fluss Staudinger, V.
NCSU 36699	NCSU	female	COLOMBIA Putumayo PNN La Paya Cabana La Paya 0°2'S 75°12'W 330m Malaise 24-25.ix.2001 E. Lozano, leg. M.2074
	ZMPA	male	Bolivia Mapiri Staudinger, V.

**Table:** *Evaniscus sulcigenis* Roman, 1917

**Identifier(s)**	**Repository**	**Sex**	**Collecting event**
NCSU 9895	NHRS	male	Manaos 1914. Bosque Municipal, nordwestlich der Stadt

**Table:** *Evaniscus tibialis* Szépligeti, 1903

**Identifier(s)**	**Repository**	**Sex**	**Collecting event**
NCSU 9896	USNM	male	Tamana Caves, Trinidad B. W. I. 3-XII-66 J. Darlington I1d*, on rocks in tunnel to bat chamber
NCSU 41747	USNM	male	Tamana Caves, Trinidad B. W. I. 4-III-66 J. Darlington I1b*c*, on rocks in tunnel to bat chamber
NCSU 41746	USNM	male	Tamana Caves, Trinidad B. W. I. 24-III-66 J. Darlington I1e*, on rocks in tunnel to bat chamber
NCSU 41745	USNM	female	[no label, but based on specimen preparation and point type it appears to be from same series as NCSU 41746: Tamana Caves, Trinidad B. W. I. J. Darlington, on rocks in tunnel to bat chamber]
	HNHM	female	Merida Venezuela 539 135 Evaniscus tibialis Szepl. id.nr.015742 HNHM Hym. Coll. {labeled as”holotype”}
NCSU 67247	INPA	female	BRASIL Amazonas Manaus ZF-2 07-21.xi.1994 Arm. suspensa 1,5m, Torre JA Rafael & J Vidal col.
NCSU 67248	INPA	male	BRASIL Amazonas Manaus Reserva [Florestal Adolpho] Ducke, 26 Km NE Manaus -2.9558, -59.9222 08-10.viii.1981 arb. flight trap, 1-2m
NCSU 67249	INPA	female	BRASIL Amazonas Manaus Reserva [Florestal Adolpho] Ducke, Igarapé Tinga -2.9558, -59.9222 10-20.v.2004 Malaise AL Henriques col.
NCSU 67250	INPA	female	BRASIL Amazonas Novo Airão/Barcelos Parque Nacional do Jau, Rio Unini -1.7317, -61.9089 20-24.vi.1996 Malaise AL Henriques, J Vidal, FL Oliveira col.
NCSU 67251	INPA	male	BRASIL Amazonas Manaus ZF03 Km 23, Fazenda Estelo, Reserva 1112 (RLO) -2.4339, -59.8542 vii.1986 Malaise Bert Klein col.
NCSU 67252	INPA	male	BRASIL Amazonas Manaus ZF03 Km 23, Fazenda Estelo, Reserva 1112 (RLO) -2.3922, -59.8775 ix.1986 Malaise Bert Klein col.
NCSU 67253	INPA	female	BRASIL Amazonas Manaus ZF03 Km 23, Fazenda Estelo, Reserva 1208 (RCN) -2.3761, -59.8775 08-15.22.iv.1987 Malaise Bert Klein col.
NCSU 67256	MPEG	male	BRASIL Pará Altamira Bom Jardim -5.0010, -54.0880 07-10.viii.2008 Malaise(10)
	BMNH	male	4383 BRITISH GUYANA: Essequibo R., Moraballi Creek 18.x.1929 Oxf. Univ. Expedn. B.M. 1929-435 Evaniscus tibialis Szepligeti 1903 det. M. Huben 1995 ID# 0227.6
	BMNH	male	967 BRITISH GUYANA: Essequibo R., Moraballi Creek 27.viii.1929 Oxf. Univ. Expedn. B.M. 1929-485
NCSU 67254	INPA	male	Brazil: Pará São João do Pirabas, Japerica 16-18.xii.1992 armadilha 1.6m suspensa J Dias col.
NCSU 67255	INPA	male	Brazil: Amazonas: Manaus ZF03 Km 23, Fazenda Estelo, Reserva 1208 (RCN) 2°22’34’’S, 59°52’39’’W 08-15.iv.1987 Malaise Bert Klein col

## Appendix C

Verbose export of specimens examined.
